# Health Equity in Artificial Intelligence and Primary Care Research: Protocol for a Scoping Review

**DOI:** 10.2196/27799

**Published:** 2021-09-17

**Authors:** Jonathan Xin Wang, Sulaiman Somani, Jonathan H Chen, Sara Murray, Urmimala Sarkar

**Affiliations:** 1 Center for Vulnerable Populations at San Francisco General Hospital University of California San Francisco San Francisco, CA United States; 2 Division of General Internal Medicine University of California San Francisco San Francisco, CA United States; 3 Icahn School of Medicine at Mount Sinai New York City, NY United States; 4 Center for Biomedical Informatics Research Division of Hospital Medicine Stanford Department of Medicine Stanford, CA United States

**Keywords:** artificial intelligence, health information technology, health informatics, electronic health records, big data, data mining, primary care, family medicine, decision support, diagnosis, treatment, scoping review, health equity, health disparity

## Abstract

**Background:**

Though artificial intelligence (AI) has the potential to augment the patient-physician relationship in primary care, bias in intelligent health care systems has the potential to differentially impact vulnerable patient populations.

**Objective:**

The purpose of this scoping review is to summarize the extent to which AI systems in primary care examine the inherent bias toward or against vulnerable populations and appraise how these systems have mitigated the impact of such biases during their development.

**Methods:**

We will conduct a search update from an existing scoping review to identify studies on AI and primary care in the following databases: Medline-OVID, Embase, CINAHL, Cochrane Library, Web of Science, Scopus, IEEE Xplore, ACM Digital Library, MathSciNet, AAAI, and arXiv. Two screeners will independently review all abstracts, titles, and full-text articles. The team will extract data using a structured data extraction form and synthesize the results in accordance with PRISMA-ScR (Preferred Reporting Items for Systematic Reviews and Meta-Analyses Extension for Scoping Reviews) guidelines.

**Results:**

This review will provide an assessment of the current state of health care equity within AI for primary care. Specifically, we will identify the degree to which vulnerable patients have been included, assess how bias is interpreted and documented, and understand the extent to which harmful biases are addressed. As of October 2020, the scoping review is in the title- and abstract-screening stage. The results are expected to be submitted for publication in fall 2021.

**Conclusions:**

AI applications in primary care are becoming an increasingly common tool in health care delivery and in preventative care efforts for underserved populations. This scoping review would potentially show the extent to which studies on AI in primary care employ a health equity lens and take steps to mitigate bias.

**International Registered Report Identifier (IRRID):**

PRR1-10.2196/27799

## Introduction

Artificial intelligence (AI) is a field of computer science that aims to create systems that are capable of independent reasoning [1,2]. Despite tremendous gains in some industries, ranging from the perfection of recommendation systems [3] and optimizing supply chains [4] to self-driving cars and collaborative robotics [5], AI has continued to marginalize minority populations. One such example involves NorthPointe’s COMPAS Core solution, an algorithm that seeks to assess the risk that recent convicts would recommit a crime [6]. The algorithm has a demonstrated bias toward labeling Black individuals as being at a high risk for recommitting a crime when compared to their White counterparts, when in reality the former were half as likely to commit the crime. While this case represents a case of algorithm bias, the bias intrinsic to other AI applications may be more subtle and therefore more likely to stay undetected.

Vulnerable populations in health care, such as women and transgender individuals, Black and Latinx populations, and those with low socioeconomic status, represent cohorts of individuals who experience significant baseline health disparities and are at heightened risk of being affected by algorithmic bias [6-8]. Pre-existing and unintended biases in the development pipeline, whether they take the form of historical, representation, or aggregation bias [9], have the potential to perpetuate deeply rooted stigma, poor cohort representation, and ineffective treatment modalities in the end-product that may further discriminate against these groups through these AI systems. For example, Obermeyer et al [10] showed that a popular health care risk–scoring algorithm recommended fewer health care assessments for Black patients than for White patients, likely because the algorithm was trained from a data set where the health care system itself contained unequal access to and lower levels of care for Black patients. Such studies reflect the need for research into fairness and AI within health care.

Primary care is the cornerstone of health care delivery and serves, in theory, as the entry point for most patients into the health care setting [11]. Historically, primary care leads medicine to recognize and attend to social determinants of health, which are strong drivers of inequitable health outcomes in vulnerable populations [12,13]. Primary care includes a wide spectrum of disease and many diverse care tasks for patients, which makes augmenting clinical practice with AI tools particularly appealing and useful. Using AI for routine tasks may allow primary care clinicians to focus on complex diagnostic and therapeutic tasks and cultivate stronger patient-physician relationships [14]. To our knowledge, only 1 other scoping review has identified current AI applications in primary care [15]. We build on their work by focusing specifically on health equity. As such, this systematic scoping review aims to (1) assess the baseline representation of these vulnerable populations in the AI applications for primary care, (2) determine whether studies are cognizant of potential biases in their results, and (3) understand how, if at all, these studies address the manner in which these biases affect the model’s impact on vulnerable populations, either positively or negatively, in the primary care setting.

## Methods

### Scoping Review

We selected a scoping review as the best method for assessing the research landscape of AI and health equity in primary care because it offers a way to systematically identify key research gaps, opportunities, evidence, and concepts in this understudied space. This type of review differs from systematic reviews and meta-analyses in that it does not narrow the parameters of the review to a specific quality assessment. Instead, it is a systematic approach to examine the landscape of a research field using broad questions to examine both empirical and conceptual aspects [16,17]. This is particularly important in the fields of health equity, primary care, and AI, where much of the literature is currently focused on specific outcomes or aspects of care [18-21]. Equity considerations extend across multiple outcomes and therefore require a scoping review to draw overall conclusions. Our protocol, developed on the basis of seminal work by Arksey and O’Malley [16], includes six stages: (1) identification of the research question; (2) identification of relevant studies; (3) study selection; (4) data extraction; (5) collation, summarization, and reporting of the results; and (6) consultation of knowledge users. We followed the PRISMA-ScR (Preferred Reporting Items for Systematic Reviews and Meta-Analyses for Scoping Reviews) checklist [22] and registered our protocol with the Open Science Framework (digital object identifier: 10.17605/OSF.IO/WGSB3). To identify articles of interest, we conducted a search update on the basis of a previous study by Kueper et al [15], who conducted a systematic scoping review of AI and primary care research in May 2020.

### Step 1: Identifying the Research Question

A committee of medical professionals at different levels (medical students and attending physicians) with multiple domain expertise (AI, primary care, and fairness in machine learning) and training in recognition of health care disparities led the scope of this study. We used the methodology of Arksey and O’Malley [16] and Levac et al [23] to guide the discussions for determining the research questions we sought to investigate. We considered vulnerable populations on the basis of the PROGRESS (place of residence, race/ethnicity/culture/language, occupation, gender/sex, religion, education, socioeconomic status, and social capital) criteria [8], which include the following variables to ascertain vulnerabilities: place of residence; race, ethnicity, and culture; occupation; gender; religion; education; socioeconomic status; and social capital. We identified three key domains for assessment: representation of vulnerable populations in the underlying data set relative to the intended target population, as assessed, for example, by subgroup prevalence; author reporting of the types of bias outlined by Suresh et al [9]; and whether these studies attempt to mitigate these pre-existing biases in their systems upstream of, during, or downstream of model development (Table 1).

**Table 1:** Research questions.

Research questions	Operational definitions
What is the representation of vulnerable individuals in the intended target population for any study on artificial intelligence within primary care?	Vulnerable populations are defined as those with known disparities as described by the following categories: Place of residence (eg, rural) Race, ethnicity (eg, Black) Occupation (eg, coal miners) Gender, sex (eg, transgender) Religion (eg, Amish) Education (eg, high-school only) Socioeconomic status (eg, low income) Social capital (eg, isolation)
How well do current studies on artificial intelligence in primary care report different types of bias that may be perpetuated as health disparities by their systems?	Data extraction elements (Table 2)
What interventions do current studies on artificial intelligence in primary care use to address harmful effects of pre-existing biases in their systems?	Example interventions are listed below: Preprocessing Modified data sources Preprocessing data for fairness Model development Demographic parity Equalized odds/opportunity Disparity regularization Counterfactual fairness Postprocessing Subgroup analysis Meta-regression Quality assurance

### Steps 2 and 3: Identify Relevant Studies and Study Selection

#### Steps Overview

To guide the search strategy for our scoping review, we have developed a number of protocols and parameters. We will use Covidence [24] to manage our records and data throughout the review.

To retrieve all AI and primary care literature, we will use a similar search strategy and eligibility criteria documented by Kuepfer et al [15], in which a team of interdisciplinary experts iteratively refined search terms for 11 databases to reliably and robustly retrieve literature spanning AI and primary care globally. This study screened over 7900 articles to amass a total of 405 eligible articles at this domain intersection (Multimedia Appendix 1). Our populations of interest are vulnerable patients (who may or may not be explicitly recognized by the study of interest in our search strategy); we will include any AI intervention; the comparison will be the current standard of care without the AI intervention; and we will include any patient-level outcome of interest in primary care. Rather than combining vulnerable population search terms, we allow our search query to broadly include AI literature that addresses vulnerable populations implicitly (eg, only ensuring demographic parity for a primary clinical outcome) or, equally importantly, fails to do so at all. For example, Hannun et al [25] used a corpus of ambulatory electrocardiograhs and trained a deep learning model to predict arrhythmias, which may have strong implications of use in primary care, but provide no context on the demographic representation and comorbidity burden in their data sets. To confirm that we applied the methodology appropriately, 2 independent reviewers (JW and SS) will extract a random sample of 4% of titles from their initial search. Then, they will apply the title and abstract screening and full-text screening process, resolving disputes with a third reviewer. Cohen κ will then be calculated between the studies we select and those selected by Kueper et al [15]. This will be repeated until a Cohen κ of >0.80 is achieved. We will then include the 405 studies that were selected by Kueper et al [15].

We will also apply the search strategy and screening criteria applied to any new articles since Kueper et al [15]’s initial search on April 6, 2018 (Multimedia Appendix 1). Two independent reviewers (JW and SS) will first review all titles and abstracts on the basis of the defined eligibility criteria. Full-text versions of all identified articles will be independently reviewed by these 2 independent reviewers for inclusion after initial screening of titles and abstracts to determine whether any other further refinements to the eligibility criteria should be made. Disagreements will be resolved by an independent reviewer through discussion, and the selection process will be adjusted to reflect these subsequent changes. Articles for which no consensus can be reached will be included in the review. Based on guidelines from Cochrane Methods, the search strategy will be utilized once again if 12 months have passed since the initial search strategy and the date of publication [26].

Once this process is complete, a final PRISMA flow diagram [27] will be submitted to document the number of articles at each step of identification, screening, eligibility, and inclusion. For now, a PRISMA flow diagram containing the number of queried and screened articles is available in Multimedia Appendix 2.

#### Databases

In line with Kueper et al [15], we searched the following databases: Medline-OVID, Embase, CINAHL, Cochrane Library, Web of Science, Scopus, IEEE Xplore, ACM Digital Library, MathSciNet, AAAI, and arXiv; these will capture published studies predominantly in the fields of medicine, computer science, and the intersection of both fields.

### Step 4: Extracting the Data

We built a preliminary data framework in accordance with the suggestions of Daudt et al [28] to align data extraction with the initial research question (Table 2). One category we extracted is measuring compliance with existing AI ethics guidelines developed for the European Commission. This category was chosen after examining multiple other AI ethical guidelines, including those of the House of Lords [29] and IEEE [30]. The European Commission's guidelines were chosen because of the comprehensible key requirements, orientation toward conceptual, higher-level evaluation (rather than technical specificities), and wide adoption across the AI community [31-33]. Two authors (JW and SS) will independently extract data from the first 10 included studies and meet to determine whether the framework is specific enough for consistency and the data are sufficient for research questions outlined initially. During this process and in prior stages, it is likely that additional categories and adjustments will be made to our data extraction framework, at which time we shall consult with the research team to guide decisions on how to appropriately modify the framework. Once the reviewers reach a consensus using the data extraction framework, it will be circulated among the research team and consultation team for final comments and suggestions. Following this, additional reviewers may be brought on, in which case they are expected to match the data extracted from these first 10 included studies in order to take part in the data collection (Cohen κ>0.8). For any disputes on data extraction, a third reviewer will be involved in settling the discussion, and appropriate adjustments to the data extraction framework will be made.

**Table 2:** Data extraction elements.

Category	Elements appraised
Reviewer information	Reviewer name Reviewer comments
Bibliometrics	First and last name of the first author Title Source Year of publication Country Status of publication
Primary care function (adapted from Kueper et al [15])	Diagnostic decision support: artificial intelligence–assisted diagnostics Treatment decision support: artificial intelligence–assisted treatment, including remote management of care Referral support: artificial intelligence–assisted support for any portion of the referral process, especially for direct referrals of patients to specialist services Scheduling assistance: models for optimizing clinic schedules and overbooking Future state prediction: artificial intelligence offering predictions about the future state, such as consult service utilization or prognosis of existing conditions. (this excludes predictions of one’s chances of developing a health condition in the near term, which falls under diagnostic decision support) Health care utilization analyses: artificial intelligence extracts information retrospectively to understand more about the current processes or interactions within a health care system Knowledge base and ontology construction or use Information extraction: artificial intelligence extracts knowledge from structured or unstructured data sources Descriptive information provision: Artificial intelligence summarizes existing data in interpretable or useful ways Other: function not represented above, but specifics of function will still be recorded in case a new category emerges
Author-reported intended end-users	The intended user of the artificial intelligence product, including but not limited to patients, physicians, nurses, nurse practitioners, administrators, researchers, others, and unknown (if an end-user is not specified as the tool was still in development, a researcher was designated)
Target health condition (adapted from Kueper et al [15])	General Diabetes Cancer, non-skin Heart valves, murmurs Musculoskeletal/joint Dementia, cognitive impairment Lung apnea, chronic obstructive pulmonary disease Chronic disease, frailty Skin cancer Stroke, neurological Psychiatric Coronary artery disease Heart failure Hypertension Other cardiovascular disease Gastrointestinal/liver Ear, nose, and throat Eye and retina Trauma, emergency surgery Infection Metabolic Kidney and urinary tract Immunization, reactions Skin disorders Obesity Pediatric/developmental Other
Data set	Size: number of unique patients Time period if applicable Source of data: Electronic health record National registry Claims Remote monitoring devices (ie, smart watch or mobile phone) Other (specified) Unknown Number of institutions: single or multiple Setting (urban, rural, both, or unknown): We use the United States Census’ County Classification Lookup Table [34] to determine whether a certain area was urban or rural. If there were multiple locations, we selected both.
Compliance with “Ethics Guidelines for Trustworthy AI” [35]: which of the 7 elements were addressed (yes/no)?	Human agency and oversight: how well does the algorithm support human decision-making and permit oversight on its predictions? Technical robustness and safety: how well-suited is the algorithm for its intended use? How well does it mitigate harm? Privacy and data governance: how well does the algorithm’s data ingestion and analysis pipeline respect patient privacy (eg, HIPAA compliance) and enforce safeguards against unpermitted access? Transparency: does the artificial intelligence algorithm explain reasons for its outputs in a traceable and interpretable way? Diversity, nondiscrimination, and fairness: how biased is the algorithm with regard to its performance? How easy is it for stakeholders to provide feedback on the algorithm’s performance for its continuous development? Societal and environmental well-being: what are the societal (eg, dehumanizing relationships) and ecological (eg, energy consumption) impacts of the algorithm? Accountability: who is held responsible to ensure the algorithm’s development, outcomes, harm, and regulation?
Model fairness and focus on health equity: is the main purpose of the study specifically outlined to improve health for a vulnerable population (yes/no)?	Must be explicitly stated in the introduction or abstract as motivation for the paper to focus on at least 1 vulnerable population (though there may be other populations studied as well) defined by any of the following categories which are largely based off of the NIMHD Research Framework [36]: Place of residence (eg, rural) Race, ethnicity (eg, Black African American or Latinx) Occupation (eg, coal miners) Gender, sex (eg, transgender) Religion (eg, Amish) Education (eg, low) Socioeconomic status (eg, low income) Social capital (eg, isolation) Does the study include key variables that could reflect disparities across protected classes (eg, age, sex, or race/ethnicity)? If reported, do they include these variables in their evaluation (eg, subgroup analysis to demonstrate equal performance)? Existing biases: does the study discuss biases or potential repercussions related to vulnerable populations? [9] Historical bias (ie, data retrieval) Representation bias (ie, population representation) Measurement bias Aggregation bias Evaluation bias Deployment bias Bias mitigation: does the study attempt to reduce existing biases, either explicitly or implicitly? If so, what methodology do they employ? Preoutput (changes to the algorithm or input data) Postoutput (user education, transparency, and specifying the use case) Other
Stage of the study	Methodological development: generation of novel artificial intelligence methods or modification of existing artificial intelligence methods to accomplish a task relevant to primary care. Retrospective data analysis or model development: developed an artificial intelligence model trained on retrospectively collected data to identify trends or perform a task that awaits prospective validation. Evaluation: artificial intelligence implemented in the intended setting as part of a pilot study, such as a prospective cohort study or randomized controlled trial. Postimplementation: assessing the impact of an artificial intelligence implementation after officially deployed in its intended setting.

### Step 5: Collating, Summarizing, and Reporting the Results

Our analysis will involve both a descriptive numerical summary and an interpretive synthesis. While our approach in stage 5 will be an iterative process, we will use this section to first provide descriptive tables, frequency tables, and visual representation of the results. Further synthesis will be performed to identify current obstacles, gaps, and opportunities in the literature.

### Step 6: Consultation

Our scoping review will include consultation with other AI researchers in academia, nonprofit, and industry to enhance the perspective, applicability, and purpose of our study and ultimately offer more practical recommendations. We will engage with stakeholders at three timepoints: (1) prior to the submission of this protocol, (2) during the finalization of the data collection framework, and (3) at the end of the study during the collation, summarization, and reporting of the results.

## Results

Electronic database searches were conducted in October 2020, and title and abstract screening are currently underway. We expect to complete the remaining steps of the scoping review, including publication, by fall 2021.

## Discussion

### Principal Findings

To our knowledge, this will be the first scoping review that applies an equity lens to the existing literature on AI in primary care. Primary care has a large potential to reduce costs and improve quality of life, especially for underserved populations [37]. Many experts have lauded AI’s potential to affect primary care [14] and issues in vulnerable patient care management. By understanding AI’s current place in primary care through the lens of health care equity, researchers can develop AI interventions that address the field’s existing gaps and opportunities.

After completing this scoping review, we will write a briefing paper to address the implications of the findings in a narrative. We will also develop a manuscript and PRISMA-ScR checklist to submit for publication.

### Limitations

Our scoping review will not incorporate a peer review process for our search strategy despite being recommended in Peer Review of Electronic Search Strategies [38]. This is typically conducted for systematic reviews rather than for scoping reviews and is not feasible with the time and resource constraints we have to achieve this review [23]. Additionally, we do not engage with community members or underserved populations themselves for consultation or feedback. We believe this is important for any study related to health equity as it improves the quality and applicability of studies for the populations they hope to serve [23,39]. However, identifying and consulting with these groups has been difficult and costly to incorporate into the protocol, which has been a recurring problem in this field of research. Instead, we rely on expert stakeholders to guide our critical appraisal of the existing literature. Considering the design of this study, we also will not conduct a rigorous assessment of the included articles beyond an inequity lens [16]. Additionally, scoping reviews do not provide a clear understanding of the efficacy of current interventions in practice as systematic reviews do, which is offset by the benefit of providing breadth from a large number of studies [16]. We also limit our work to English language articles, and no proprietary research is captured in this review.

### Conclusions

AI has immense potential to improve the patient-physician relationship by augmenting physician capabilities. Primary care is an especially viable area for the integration of AI, given its early entry point, broad scope of vulnerable populations, the heavy toll these socioeconomic factors have on patient care, and the need to address these factors to manage disease more effectively. However, algorithms are susceptible to performance disparities across different subgroups, which may further reinforce pre-existing health inequities if not rigorously assessed before deployment. With this scoping review protocol, we aim to provide a process to assess the state of AI in primary care for vulnerable populations.

## Appendix

Multimedia Appendix 1 Search strategy and overview for Kueper et al [<xref ref-type="bibr" rid="ref15">15</xref>].

Multimedia Appendix 2 PRISMA (Preferred Reporting Items for Systematic Reviews and Meta-analyses) flow diagram from Kueper et al [<xref ref-type="bibr" rid="ref15">15</xref>].

## Abbreviations

AI: artificial intelligence
PRISMA-ScR: Preferred Reporting Items for Systematic Reviews and Meta-Analyses Extension for Scoping Reviews.

## References

[1] Kok J (2009). Artificial Intelligence.

[2] Baştanlar Y, Özuysal M (2014). Introduction to Machine Learning. miRNomics: MicroRNA Biology and Computational Analysis.

[3] Covington P, Adams J, Sargin E (2016). Deep Neural Networks for YouTube Recommendations.

[4] Carbonneau R, Laframboise K, Vahidov R (2008). Application of machine learning techniques for supply chain demand forecasting. Eur J Oper Res.

[5] Pierson HA, Gashler MS (2017). Deep learning in robotics: a review of recent research. Adv Robot.

[6] Rudin C, Ustun B (2018). Optimized Scoring Systems: Toward Trust in Machine Learning for Healthcare and Criminal Justice. Interfaces.

[7] Frohlich KL, Potvin L (2008). Transcending the known in public health practice: the inequality paradox: the population approach and vulnerable populations. Am J Public Health.

[8] O'Neill J, Tabish H, Welch V, Petticrew M, Pottie K, Clarke M, Evans T, Pardo Pardo J, Waters E, White H, Tugwell P (2014). Applying an equity lens to interventions: using PROGRESS ensures consideration of socially stratifying factors to illuminate inequities in health. J Clin Epidemiol.

[9] Suresh H, Guttag JV A Framework for Understanding Sources of Harm throughout the Machine Learning Life Cycle. arXiv..

[10] Obermeyer Z, Powers B, Vogeli C, Mullainathan S (2019). Dissecting racial bias in an algorithm used to manage the health of populations. Science.

[11] Donaldson MS, Yordy KD, Lohr KN, Vanselow NA (1996). Defining Primary Care. Primary Care: America's Health in a New Era.

[12] Katz A, Chateau D, Enns JE, Valdivia J, Taylor C, Walld R, McCulloch S (2018). Association of the Social Determinants of Health With Quality of Primary Care. Ann Fam Med.

[13] Khanassov V, Pluye P, Descoteaux S, Haggerty JL, Russell G, Gunn J, Levesque J (2016). Organizational interventions improving access to community-based primary health care for vulnerable populations: a scoping review. Int J Equity Health.

[14] Lin SY, Mahoney MR, Sinsky CA (2019). Ten Ways Artificial Intelligence Will Transform Primary Care. J Gen Intern Med.

[15] Kueper JK, Terry AL, Zwarenstein M, Lizotte DJ (2020). Artificial Intelligence and Primary Care Research: A Scoping Review. Ann Fam Med.

[16] Arksey H, O'Malley L (2005). Scoping studies: towards a methodological framework. Int J Soc Res Methodol.

[17] Graham ID (2012). Knowledge synthesis and the Canadian Institutes of Health Research. Syst Rev.

[18] Entezarjou A, Bonamy AE, Benjaminsson S, Herman P, Midlöv P (2020). Human- Versus Machine Learning-Based Triage Using Digitalized Patient Histories in Primary Care: Comparative Study. JMIR Med Inform.

[19] Weng SF, Reps J, Kai J, Garibaldi JM, Qureshi N (2017). Can machine-learning improve cardiovascular risk prediction using routine clinical data?. PLoS One.

[20] Zhou S, Fernandez-Gutierrez F, Kennedy J, Cooksey R, Atkinson M, Denaxas S, Siebert S, Dixon WG, O'Neill TW, Choy E, Sudlow C, Brophy S, UK Biobank Follow-up and Outcomes Group (2016). Defining Disease Phenotypes in Primary Care Electronic Health Records by a Machine Learning Approach: A Case Study in Identifying Rheumatoid Arthritis. PLoS One.

[21] Abràmoff MD, Lavin PT, Birch M, Shah N, Folk JC (2018). Pivotal trial of an autonomous AI-based diagnostic system for detection of diabetic retinopathy in primary care offices. NPJ Digit Med.

[22] Tricco AC, Lillie E, Zarin W, O'Brien KK, Colquhoun H, Levac D, Moher D, Peters MDJ, Horsley T, Weeks L, Hempel S, Akl EA, Chang C, McGowan J, Stewart L, Hartling L, Aldcroft A, Wilson MG, Garritty C, Lewin S, Godfrey CM, Macdonald MT, Langlois EV, Soares-Weiser K, Moriarty J, Clifford T, Tunçalp Ö, Straus SE (2018). PRISMA Extension for Scoping Reviews (PRISMA-ScR): Checklist and Explanation. Ann Intern Med.

[23] Levac D, Colquhoun H, O'Brien KK (2010). Scoping studies: advancing the methodology. Implement Sci.

[24] Better systematic review management. Covidence.

[25] Hannun AY, Rajpurkar P, Haghpanahi M, Tison GH, Bourn C, Turakhia MP, Ng AY (2019). Cardiologist-level arrhythmia detection and classification in ambulatory electrocardiograms using a deep neural network. Nat Med.

[26] Methodological Expectations of Cochrane Intervention Reviews. Cochrane Methods.

[27] Moher D, Liberati A, Tetzlaff J, Altman DG, PRISMA Group (2009). Preferred reporting items for systematic reviews and meta-analyses: the PRISMA statement. PLoS Med.

[28] Daudt H, van Mossel C, Scott S (2013). Enhancing the scoping study methodology: a large, inter-professional team's experience with Arksey and O'Malley's framework. BMC Med Res Methodol.

[29] (2017). Artificial Intelligence Committee. AI in the UK: ready, willing and able?. UK Parliament.

[30] IEEE Ethics in Action in Autonomous and Intelligent Systems. IEEE.

[31] Hagendorff T (2020). The Ethics of AI Ethics: An Evaluation of Guidelines. Minds Mach.

[32] Rességuier A, Rodrigues R (2020). AI ethics should not remain toothless! A call to bring back the teeth of ethics. Big Data Soc.

[33] Jobin A, Ienca M, Vayena E (2019). The global landscape of AI ethics guidelines. Nat Mach Intell.

[34] Urban and Rural. US Census Bureau.

[35] Communication: Building Trust in Human Centric Artificial Intelligence. European Commission.

[36] Overview. National Institute of Minority Health and Health Disparities.

[37] Morelli V (2017). An Introduction to Primary Care in Underserved Populations: Definitions, Scope, and Challenges. Prim Care.

[38] McGowan J, Sampson M, Salzwedel DM, Cogo E, Foerster V, Lefebvre C (2016). PRESS Peer Review of Electronic Search Strategies: 2015 Guideline Statement. J Clin Epidemiol.

[39] Kilbourne AM, Switzer G, Hyman K, Crowley-Matoka M, Fine MJ (2006). Advancing health disparities research within the health care system: a conceptual framework. Am J Public Health.

