# Supplementary material for: Health Equity in Artificial Intelligence and Primary Care Research: Protocol for a Scoping Review
Source: JMIR Res Protoc. 2021 Sep 17;10(9):e27799. doi: 10.2196/27799 (PMC8486995; doi:10.2196/27799)
Supplement: Multimedia Appendix 1 [file resprot_v10i9e27799_app1.pdf]

Supplemental Material for:

Kueper JK, Terry AL, Zwarenstein M, Lizotte DJ. Artificial intelligence and primary care research: a scoping review. *Ann Fam Med*. 2020;18(3):250-258.

SUPPLEMENTAL APPENDIX 1: PRISMA-ScR

Preferred Reporting Items for Systematic reviews and Meta-Analyses extension for Scoping Reviews (PRISMA-ScR) Checklist

SECTION	ITEM	PRISMA-ScR CHECKLIST ITEM	REPORTED ON PAGE #
**TITLE**			
Title	1	Identify the report as a scoping review.	1
**ABSTRACT**			
Structured summary	2	Provide a structured summary that includes (as applicable): background, objectives, eligibility criteria, sources of evidence, charting methods, results, and conclusions that relate to the review questions and objectives.	2
**INTRODUCTION**			
Rationale	3	Describe the rationale for the review in the context of what is already known. Explain why the review questions/objectives lend themselves to a scoping review approach.	1-2
Objectives	4	Provide an explicit statement of the questions and objectives being addressed with reference to their key elements (e.g., population or participants, concepts, and context) or other relevant key elements used to conceptualize the review questions and/or objectives.	2
**METHODS**			
Protocol and registration	5	Indicate whether a review protocol exists; state if and where it can be accessed (e.g., a Web address); and if available, provide registration information, including the registration number.	2
Eligibility criteria	6	Specify characteristics of the sources of evidence used as eligibility criteria (e.g., years considered, language, and publication status), and provide a rationale.	2,3
Information sources*	7	Describe all information sources in the search (e.g., databases with dates of coverage and contact with authors to identify additional sources), as well as the date the most recent search was executed.	2
Search	8	Present the full electronic search strategy for at least 1 database, including any limits used, such that it could be repeated.	Supplemental Appendix 2
Selection of sources of evidence†	9	State the process for selecting sources of evidence (i.e., screening and eligibility) included in the scoping review.	2,3
Data charting process‡	10	Describe the methods of charting data from the included sources of evidence (e.g., calibrated forms or forms that have been tested by the team before their use, and whether data charting was done independently or in duplicate) and any	3

SECTION	ITEM	PRISMA-ScR CHECKLIST ITEM	REPORTED ON PAGE #
		processes for obtaining and confirming data from investigators.	
Data items	11	List and define all variables for which data were sought and any assumptions and simplifications made.	3, Supplemental Appendix 3
Critical appraisal of individual sources of evidence§	12	If done, provide a rationale for conducting a critical appraisal of included sources of evidence; describe the methods used and how this information was used in any data synthesis (if appropriate).	NA
Synthesis of results	13	Describe the methods of handling and summarizing the data that were charted.	3
**RESULTS**			
Selection of sources of evidence	14	Give numbers of sources of evidence screened, assessed for eligibility, and included in the review, with reasons for exclusions at each stage, ideally using a flow diagram.	3, Figure 1
Characteristics of sources of evidence	15	For each source of evidence, present characteristics for which data were charted and provide the citations.	3-5; Table 1; Figures 2 – 4, Supplemental Appendix 3
Critical appraisal within sources of evidence	16	If done, present data on critical appraisal of included sources of evidence (see item 12).	NA
Results of individual sources of evidence	17	For each included source of evidence, present the relevant data that were charted that relate to the review questions and objectives.	NA (too many studies to do in a meaningfully interpretable way)
Synthesis of results	18	Summarize and/or present the charting results as they relate to the review questions and objectives.	3-5; Table 1; Figures 2 – 4; Supplemental Appendix 3
**DISCUSSION**			
Summary of evidence	19	Summarize the main results (including an overview of concepts, themes, and types of evidence available), link to the review questions and objectives, and consider the relevance to key groups.	5,6
Limitations	20	Discuss the limitations of the scoping review process.	6
Conclusions	21	Provide a general interpretation of the results with respect to the review questions and objectives, as well as potential implications and/or next steps.	5,6,7
**FUNDING**			
Funding	22	Describe sources of funding for the included sources of evidence, as well as sources of funding for the scoping review. Describe the role of the funders of the scoping review.	7

JBI = Joanna Briggs Institute; PRISMA-ScR = Preferred Reporting Items for Systematic reviews and Meta-Analyses extension for Scoping Reviews.
* Where *sources of evidence* (see second footnote) are compiled from, such as bibliographic databases, social media platforms, and Web sites.

† A more inclusive/heterogeneous term used to account for the different types of evidence or data sources (e.g., quantitative and/or qualitative research, expert opinion, and policy documents) that may be eligible in a scoping review as opposed to only studies. This is not to be confused with *information sources* (see first footnote).
‡ The frameworks by Arksey and O'Malley (6) and Levac and colleagues (7) and the JBI guidance (4, 5) refer to the process of data extraction in a scoping review as data charting.
§ The process of systematically examining research evidence to assess its validity, results, and relevance before using it to inform a decision. This term is used for items 12 and 19 instead of "risk of bias" (which is more applicable to systematic reviews of interventions) to include and acknowledge the various sources of evidence that may be used in a scoping review (e.g., quantitative and/or qualitative research, expert opinion, and policy document).

From: Tricco AC, Lillie E, Zarin W, O'Brien KK, Colquhoun H, Levac D, et al. PRISMA Extension for Scoping Reviews (PRISMA-ScR): Checklist and Explanation. Ann Intern Med. ;169:467–473. doi: 10.7326/M18-0850

SUPPLEMENTAL APPENDIX 2: SEARCH STRATEGIES

Figure S1: Development of search strategies

Identify documents to inform search strategy

- PubMed Search using Medical Subject Headings for AI and PC (n=88).
- Harvest subject headings and keywords from relevant documents (n=7).

Develop initial search strategy

- Use harvested terms to inform comprehensive search strategy in conjunction with topic area knowledge and discussion amongst reviewers.

Pilot test search strategy in health and computer science databases

- Revise strategy in an iterative fashion to balance comprehensiveness with feasibility.
    - For example, due to subject-area terminology differences, certain terms were used exclusively in health sciences or in computer science databases (e.g. “knowledge base”).

Test search strategy comprehensiveness

- All relevant documents from the initial search were re-identified using the final search strategy in Medline-OVID.
- Final revisions were made as necessary for remaining databases.
- Assessed amount of literature missed by restricting to English language.

Databases used:

i. Medline-OVID
ii. EMBASE
iii. Cinahl
iv. Cochrane Library
v. Web of Science
vi. Scopus
vii. IEEE Xplore
viii. ACM Digital Library
ix. MathSciNet
x. AAAI (https://aaai.org/ocs/index.php/index/index/search/advanced)
xi. arXiv

Database Searching Notes

The databases listed above have different search capabilities in terms of keywords and subject headings. We used the most rigorous approach possible for each database, whereby approaches can be broken down into three general categories:

1) **Search with keywords and subject headings:**

- Medline-OVID
  - Keywords were used to search title, abstract, and author keywords.
- Embase
  - Keywords were used to search title, abstract, and author keywords.
- Cinahl
  - Keywords were used to search title and abstract.
- Cochrane
  - Keywords were used to search title, abstract, and keywords.
- ACM Digital Library
  - No wildcard (*), use full spellings.
  - Only used artificial intelligence subject headings (CCS); health related headings are too broad and captured too many irrelevant documents to maintain review feasibility.
  - Search "The ACM Full-Text Collection."

Search strategy for category 1:

i) Keywords and subject headings for artificial intelligence concept were searched with OR.
ii) Keywords and subject headings for primary care concept were searched with OR.
iii) i) and ii) were combined with AND.

2) Search with keywords only:

- Web of Science
  - Keywords in "Topic" field were used to search title, abstract, author keywords, and keywords plus.
- Scopus
  - Keywords were used to search title, abstract, and keywords.
- MathSciNet
  - Keywords in 'Anywhere' field were used to search author, author/related, title, review text, journal, institution code, series, MSC primary/secondary, MSC primary, MR number, and reviewer.
- arXiv
  - arXiv API was accessed using python.
  - Keywords in 'all' field were used to search title, author, abstract, comment, journal reference, subject category, report number, and id.

Search strategy for category 2:

i) Keywords for artificial intelligence concept were searched with OR.
ii) Keywords for primary care concept were searched with OR.
iii) i) and ii) were combined with AND.

3) Search with limited keywords only:

- IEEE Xplore
  - Limited to 12 keywords.
  - Keywords were used to search metadata (abstract, index terms, bibliographic citation data.)
  - No wildcard (*) within phrase searching, so we wrote out the 12 terms in full.
- AAAI
  - Limited to 254 characters.

- Used only primary care concept keywords because redundant to search artificial intelligence terms in artificial intelligence proceedings.
- Case sensitive; spelled out keywords that are most important and most likely to be capitalized differentially by different authors using upper and lower case first letter(s) and spelled out less important or less capitalization-ambiguous keywords using only lower case.

Search strategy for IEEE Xplore:

i) Keywords for artificial intelligence concept were searched with OR.
ii) Keywords for primary care concept were searched with OR.
iii) i) and ii) were combined with AND.

Search strategy for AAAI:

i) Keywords for primary care concept were searched with OR.

**Search Terms for Health Sciences Databases.**					
**Concept**	**Key Words**<br><br>(syntax for Medline-OVID and EMBASE)	**Medline-Ovid**	**EMBASE**	**CINAHL**	**Cochrane Library**<br><br>(default explodes subject headings)
**Artificial Intelligence**	(Artificial Intelligence OR Computer Heuristics OR Expert System* OR Fuzzy Logic OR Machine Learning OR Support Vector Machine OR Natural Language Processing OR Neural Network* OR Robotic* OR	exp Artificial Intelligence/ OR Data Mining/ OR exp Decision Making, Computer Assisted/ OR exp Decision Support Techniques/	Exp Artificial Intelligence/ OR Expert System/ OR Fuzzy Logic/ OR Exp Machine Learning/ OR Natural Language Processing/ OR Robotics/ OR Computer Assisted Diagnosis/ OR Exp Computer Assisted Therapy/ OR Knowledge Base/ OR Knowledge	(MH “Artificial Intelligence+”) OR (MH “Data Mining”) OR (MH "Decision Making, Computer Assisted") OR (MH "Diagnosis, Computer Assisted+") OR (MH "Therapy,	[mh “Artificial Intelligence”] OR [mh “Decision Making, Computer Assisted”] OR [mh “Decision Support Techniques”] OR [mh “Data Mining” not exploded; separate line]

	Deep Learning OR Knowledge Representation OR Automated Reasoning OR Computer Vision OR Data Mining OR Bayesian Network* OR Bayes Network*).ti,ab,kw.		Base/ OR Ontology Development/	Computer Assisted+") OR (MH "Decision Support Techniques+")	
**Primary Care**	(Primary Care OR Primary Health Care OR Primary Healthcare OR Primary Medical Care OR Family Medicine OR Family Healthcare OR Family Health Care OR Family Physician* OR Family Pract* OR General Practitioner* OR Nurse Practitioner* OR Family Doctor* OR Family Nurse* OR Community Medicine OR Community Pract*	Primary Health Care/ OR Physicians, Family/ OR Physicians, Primary Care/ OR General Practitioners/ OR exp General Practice/ OR Community Medicine/ OR Nurse Practitioners/ OR Family Nurse Practitioners/ OR Primary Care Nursing/ OR Nurses, Community Health/ OR Ambulatory Care/	Exp Primary Health Care/ OR Family Medicine/ OR Community Medicine/ OR Family Health/ OR General Practitioner/ OR General Practice/ OR Ambulatory Care/ OR Ambulatory Care Nursing/ OR Nurse Practitioner/ OR Family Nurse Practitioner/	(MH “Primary Health Care”) OR (MH “Physicians, Family”) OR (MH “Family Practice”) OR (MH “Community Medicine”) OR (MH “Community Health Centers”) OR (MH “Nurse Practitioners”) OR (MH “Family Nurse Practitioners”) OR (MH “Ambulatory Care”) OR (MH	[mh “Primary Health Care”] OR [mh “Physicians, Primary Care”] OR [mh “Primary Care Nursing”] OR [mh “Physicians, Family”] OR [mh “General Practitioners”] OR [mh “General Practice”] OR [mh “Community Medicine”] OR [mh “Nurse Practitioners”] OR [mh “Family Nurse Practitioners”] OR [mh “Nurses, Community Health”] OR [mh “Ambulatory Care”]

	OR Ambulatory Care).ti,ab,kw.			"Ambulatory Care Nursing") OR (MH "Ambulatory Care Facilities")	

Note: Keywords from the above ("health sciences") databases were used for Scopus and Web of Science.

**Search Terms for Computer Science Databases**		
**Concept**	**Key Words** (MathSciNet syntax)	**ACM Digital Library**
**Artificial Intelligence**	("Artificial Intelligence" OR "Computer Heuristics" OR "Expert System*" OR "Fuzzy Logic" OR "Knowledge Base" OR "Machine Learning" OR "Natural Language Processing" OR "Support Vector Machine" OR "Neural Network*" OR "Robotic*" OR "Deep Learning" OR "Knowledge Representation" OR "Automated Reasoning" OR "Computer Vision" OR "Data Mining" OR "Bayesian Network*" OR "Bayes Network*")	"Artificial Intelligence" "Robotic Planning" "Distributed Artificial Intelligence" "Computer Vision" "Machine Learning" "Machine Learning Algorithms"
**Primary Care**	("Primary Care" OR "Primary Health Care" OR "Primary Healthcare" OR "Primary Medical Care" OR "Family Medicine" OR "Family Healthcare" OR "Family Health Care" OR "Family Physician*" OR "Family	None.

	Pract*" OR "General Practitioner*" OR "Nurse Practitioner*" OR "Family Doctor*" OR "Family Nurse*" OR "Community Medicine" OR “Community Pract*” OR "Ambulatory Care")	

Additional Search Strings:

CINAHL & Cochrane Library keyword syntax (all Table 1 keywords) to be combined with subject headings:

(“Artificial Intelligence” OR “Computer Heuristics” OR “Expert System*” OR “Fuzzy Logic” OR “Machine Learning” OR “Support Vector Machine” OR “Natural Language Processing” OR “Neural Network*” OR “Robotic*” OR “Deep Learning” OR “Knowledge Representation” OR “Automated Reasoning” OR “Computer Vision” OR “Data Mining” OR “Bayesian Network*” OR “Bayes Network*”)

(“Primary Care” OR “Primary Health Care” OR “Primary Healthcare” OR “Primary Medical Care” OR “Family Medicine” OR “Family Healthcare” OR “Family Health Care” OR “Family Physician*” OR “Family Pract*” OR “General Practitioner*” OR “Nurse Practitioner*” OR “Family Doctor*” OR “Family Nurse*” OR “Community Medicine” OR “Community Pract*” OR “Ambulatory Care”)

Web of Science syntax (use advanced search page; all Table 1 keywords):

Line 1: TS=(“Artificial Intelligence” OR “Computer Heuristics” OR “Expert System*” OR “Fuzzy Logic” OR “Machine Learning” OR “Support Vector Machine” OR “Natural Language Processing” OR “Neural Network*” OR “Robotic*” OR “Deep Learning” OR “Knowledge Representation” OR “Automated Reasoning” OR “Computer Vision” OR “Data Mining” OR “Bayesian Network*” OR “Bayes Network*”) *AND* **LANGUAGE:** (English)

Line 2: TS=(“Primary Care” OR “Primary Health Care” OR “Primary Healthcare” OR “Primary Medical Care” OR “Family Medicine” OR “Family Healthcare” OR “Family Health Care” OR “Family Physician*” OR “Family Pract*” OR “General

Practitioner*” OR “Nurse Practitioner*” OR “Family Doctor*” OR “Family Nurse*” OR “Community Medicine” OR “Community Pract*” OR “Ambulatory Care”) *AND* **LANGUAGE:** (English)

Line 3: #2 AND #1

Scopus search syntax (all Table 1 keywords):

(TITLE-ABS-KEY ((“Artificial Intelligence” OR “Computer Heuristics” OR “Expert System*” OR “Fuzzy Logic” OR “Machine Learning” OR “Support Vector Machine” OR “Natural Language Processing” OR “Neural Network*” OR “Robotic*” OR “Deep Learning” OR “Knowledge Representation” OR “Automated Reasoning” OR “Computer Vision” OR “Data Mining” OR “Bayesian Network*” OR “Bayes Network*”) AND (“Primary Care” OR “Primary Health Care” OR “Primary Healthcare” OR “Primary Medical Care” OR “Family Medicine” OR “Family Healthcare” OR “Family Health Care” OR “Family Physician*” OR “Family Pract*” OR “General Practitioner*” OR “Nurse Practitioner*” OR “Family Doctor*” OR “Family Nurse*” OR “Community Medicine” OR “Community Pract*” OR “Ambulatory Care”))) AND (LIMIT-TO (LANGUAGE, “English”))

ACM Digital Libraries syntax (all Table 2 keywords and subject headings):

+("Artificial Intelligence" "Computer Heuristics" "Expert Systems" "Fuzzy Logic" "Knowledge Base" "Machine Learning" "Natural Language Processing" "Support Vector Machine" "Neural Network" "Robotic" "Deep Learning" "Knowledge Representation" "Automated Reasoning" "Computer Vision" "Bayesian Network" "Bayes Network" (+acmdlCCS:(“Artificial Intelligence” “Robotic planning” “Distributed Artificial Intelligence” “Computer Vision” “Machine Learning” “Machine Learning Algorithms”)))
+("Primary Care" "Primary Health Care" "Primary Healthcare" "Primary Medical Care" "Family Medicine" "Family Healthcare" "Family Health Care" "Family Physician" "Family Practice" "Family Practitioner" "General Practitioner" "Nurse Practitioner" "Community Medicine" "Community Practice" "Ambulatory Care" "Family Doctor" "Family Nurse")

IEEE syntax (use Command Search, metadata only; subset of Table 2 keywords (database limit is 12)):

((“Artificial Intelligence” OR “Machine Learning” OR “Data Mining” OR “Natural Language Processing”) AND (“Primary Care” OR “Primary Health Care” OR “Primary Healthcare” OR “Family Physician” OR “General Practitioner” OR “Family Doctor” OR “Nurse Practitioner” OR “Family Medicine”))

AAAI syntax (use 'search all categories for" line at https://aaai.org/ocs/index.php/index/index/search/advanced; subset of Table 2 keywords (254 character limit)):
"Primary Care" OR "primary care" OR "Primary Health Care" OR "primary health care" OR "Primary Healthcare" OR "primary healthcare" OR "family physician" OR "general practitioner" OR "family doctor" OR "nurse practitioner" OR "family medicine"

arXiv API access python code (adapted from https://arxiv.org/help/api/user-manual#Architecture; all Table 2 keywords):

```
import urllib
url =
'https://export.arxiv.org/api/query?search_query=all:%28%22artificial+intelligence%22+OR+%22computer+heuristics%22+OR+%22expert+system*%22+OR+%22fuzzy+logic%22+OR+%22knowledge+base%22+OR+%22machine+learning%22+OR+%22natural+language+processing%22+OR+%22support+vector+machine%22+OR+%22neural+network*%22+OR+%22robotic*%22+OR+%22deep+learning%22+OR+%22knowledge+representation%22+OR+%22automated+reasoning%22+OR+%22computer+vision%22+OR+%22data+mining%22+OR+%22bayesian+network*%22+OR+%22bayes+network*%22%29+AND+all:%28%22primary+care%22+OR+%22primary+health+care%22+OR+%22primary+healthcare%22+OR+%22primary+medical+care%22+OR+%22family+medicine%22+OR+%22family+healthcare%22+OR+%22family+health+care%22+OR+%22family+physician*%22+OR+%22family+pract*%22+OR+%22general+practitioner*%22+OR+%22nurse+practitioner*%22+OR+%22family+doctor*%22+OR+%22family+nurse*%22+OR+%22community+medicine%22+OR+%22community+pract*%22+OR+%22ambulatory+care%22%29&start=0&max_results=2000'
data = urllib.urlopen(url).read()
print data
```

SUPPLEMENTAL APPENDIX 3: ADDITIONAL METHODS & RESULTS

Table 1S: Data Extraction Field Characterizations.

**Field**	**Definition and Subfields**
Citation Information	Last and first name of first author, year of publication, and title
Study Purpose(s)	Three mutually exclusive overall research purposes for AI in the study:<br><br>1. *Method Development/Adaptation:* Research that created novel AI methods or modified existing AI methods to accomplish a task relevant to PC. For example, developing a new supervised machine learning algorithm to learn a model that will predict the probability of pathological heart murmurs using digital heart sound recording data.[78] This category includes studies that compare the performance of AI methods to the performance of humans or that include consultation with end users to inform tool development, as this is considered part of model testing, which may lead to further modifications before evaluating performance in the setting that the AI is intended to support (e.g. clinical practice.)<br><br>2. *Data Analysis:* Existing AI methods were used to analyze and/or extract information from data. For example, using natural language processing algorithms to identify cases of familial hypercholesteremia from electronic health records [79].<br><br>3. *Evaluation:* Research that included AI implemented in its intended setting, possibly as part of a pilot study to assess impact or usability characteristics of a tool. For example, assessing the impact of a machine learning-derived diagnosis model on reducing cervical intraepithelial neoplasia overdiagnosis in a Dutch national population screening program [80].
Author Appointment(s)	Author affiliations as presented on the manuscript, divided into 16 categories: *Biological and Biomedical Sciences, Company, Computer Science, Engineering, Epidemiology and Biostatistics, Health Sciences, Informatics, Mathematics, Medicine – Unspecified, Medicine – Specialty, Family Medicine and Primary Care, Nursing, Public Health, Statistics, Other (specified),* and *Unknown* when not enough affiliation information was provided to identify a broad discipline. When an author had multiple affiliations, all were recorded.
Primary Care Function(s)	Nine categories of PC functions or tasks that the researched AI supported or is intended to support in the future:

	1. *Diagnostic Decision Support*: AI provided information to inform diagnosis, such as the probability that a patient has a particular condition.<br><br>2. *Treatment Decision Support*: AI provided information to inform treatment decisions, whereby treatment was interpreted broadly to include any management or care provided (or absence of unnecessary actions) to someone with the health condition(s) or symptom(s) of interest.<br><br>3. *Referral Support*: AI provided information to support decisions about referring patients to specialist services or AI assisted with technical aspects of the referral process.<br><br>4. *Future State Prediction*: AI provided predictions towards future events, for example utilization of emergency department, development of a health condition, or prognosis for an existing condition.<br><br>5. *Health Care Utilization Analyses*: AI provided information about interactions with or processes within health care systems, for example frequency or quantity of patient visits.<br><br>6. *Knowledge Base and Ontology Construction or Use:* Construction or use of knowledge bases or ontologies including PC concepts.<br><br>7. *Information Extraction*: AI used to extract knowledge from structured or unstructured data (e.g. electronic medical records) for further use.<br><br>8. *Descriptive Information Provision*: AI used to summarize data in a meaningful way for human interpretation, for example prevalence of a condition or patterns of patient profiles.<br><br>9. *Other (specified)*: The PC function was not represented by the above categories; specifics were recorded.
Author Reported Intended End-User(s)	People who the research or research end-product was stated as intended for, regardless of whether those intended end users were involved with the research or how close the research was to being applicable for those users in practice setting: *Patient, Physician, Nurse, Nurse Practitioner, Administrator, Researcher, Other (specified), or Unknown*. If the study was developing a deployable AI method or tool (broadly defined) but more research was needed before

	the AI method of interest would be ready to implement or be utilized by its intended end user, *Researcher* was included as a target end user.
Target Health Condition(s)	The health condition of interest as stated by the study authors or inferred by reviewers, or *Unknown* if no condition was stated or inferable. Conditions were extracted in full form and MZ later organized them into 27 and 10 category formats. When a study intended for AI to be applicable for all health conditions "*General*" was used; specifics about any test conditions were also extracted.
Location of Data Source(s) or intended location of implementation	Country or next level of granularity where data were collected, or the geographical location where the study stated implementation would occur. *Unknown* was used when the location of data source was not stated or when all data were simulated.
Subfield(s) of Artificial Intelligence	Artificial Intelligence methods were organized according to 10 subfields; a single study may include one or more subfields:<br><br>1. *Bayesian Network:* Graphical models (directed acyclic graphs) used to describe dependency relationships among variables that enable the efficient representation of multivariate probability distributions. The resulting distributions can be queried to find the probability of an event occurring given a particular set of evidence. Bayesian networks can be developed manually, such as from physician input, learned from data, or created using a combination of the two. For example, Teles et al. (2015) use a Bayesian Network to assist the diagnosis of dengue fever disease. The model includes variables for dengue fever risk factors, such as 'Respiratory Distress'. For prediction, a person's current risk factor variable values are inputted and the conditional probability they have dengue fever is outputted.[81]<br><br>2. *Computer Vision:* Includes extracting visual information and understanding it. Computer vision is distinct from image processing, which includes modifying an existing or creating a new image without focusing on the meaning of the image. For example, Zouridakis et al. (2015) present a smartphone app whereby a picture of a skin lesion is taken and computer vision is used to interpret the image and assess the likelihood of malignancy.[82]<br><br>3. *Data Mining:* The process of eliciting information from collections of data, such as by finding and counting pattern occurrences using inferential algorithms; humans may then interpret these patterns. For example, Soler et al. (2015) used data mining on electronic medical records to identify relationships between reasons for encounter and diagnoses recorded for the corresponding visit.[83] We did not consider

	extracting information in a structured way, such as using a database query to get a basic count of disease X diagnoses, to be the type of data mining that falls under the umbrella of artificial intelligence.<br><br>4. *Expert System:* Consists of two parts: 1) a knowledge base that contains facts and rules, such as if-then statements derived from medical guidelines and 2) an inference engine that uses the knowledge base to arrive at conclusions or answers to questions. For example, Lange et al. (1997) demonstrate the use of an expert system called Iliad for teaching diagnostic reasoning to Nurse Practitioner students.[84] Illiad's knowledge base is made up of medical facts and relationships. Bayesian or probabilistic and Boolean or deterministic reasoning may be used with the knowledge base to arrive at a level of confidence about a diagnosis.[84]<br><br>5. *Fuzzy Models:* Rely on fuzzy logic and fuzzy sets to represent problems with uncertainty. They are often used to provide more flexibility to outcomes instead of requiring strict classification into pre-defined groups. For example, Katigari et al. (2017) used a fuzzy model as the inference engine for an expert system designed to support diagnosis of diabetic neuropathy.[85] Model input includes parameters such as time with diabetes, symptom severity, and laboratory blood test values; model output is an estimate of diabetic neuropathy severity.[85]<br><br>6. *Natural Language Processing:* The ability to read language used by humans and interpret it in a meaningful way; this is often accomplished by analysing syntactic and semantic characteristics of language. The input language may be audio or written. For example, Koeling et al. (2011) used natural language processing to analyse free text portions of medical records and enhance the accuracy of ovarian cancer symptom detection compared to only using the structured portion of medical records.[86]<br><br>7. *Robotics:* Robotics within artificial intelligence refers to machines that can act autonomously to navigate and alter their environment. A robot may rely on other types of artificial intelligence, such as computer vision and natural language processing, to accomplish this. Robotics outside of artificial intelligence include machines that are programmed by humans to perform a defined set of actions. No examples of robotics were captured by our review.

	8. *Supervised Machine Learning:* Involves an algorithm learning to associate labels with observations. In the context of health, the label is often an outcome, e.g. a disease state or outcome, and the observations are often patient variables. Labels may be numeric values or categorical classifications. Supervised machine learning uses existing labelled data which contain a collection of observations together with their correct label to produce a model that is able to assign a label to new, previously unseen observations. Supervised machine learning techniques include Support Vector Machines, K-Nearest Neighbours, Naïve Bayes Classifier, and Random Forest Decision Trees. For example, Cox et al. (2016) used supervised machine learning to help identify undiagnosed post-stroke spasticity.[87] A model was trained using a large PC database that included the outcome of interest, post stroke spasticity events, and 72 candidate variables to predict the outcome, such as demographic information, prescriptions, and medical diagnoses.[87] They then used the model to identify people who had a high probability of post-stroke spasticity and checked whether the event was recorded in their records; the results of this study suggested an under recording of post stroke spasticity in PC records.[87]<br><br>9. *Unsupervised Machine Learning:* Algorithms learn patterns from unlabelled data (unlabelled meaning there are not defined, known outcome categories as was the case for supervised machine learning). Common unsupervised machine learning techniques include clustering data items into groups based on their similarity, association mining to identify observations that tend to occur together, autoencoders to condense data while maintaining adequate fidelity, and feature separation to examine different aspects of a dataset independently. For example, Newcomer et al. (2011) used cluster analysis on data from a health care organization to identify groups of complex patients who may benefit from targeted care strategies.[88]<br><br>10. *Other (specified):* There are additional types of AI not captured by the above, such as multi-agent systems. We did not expect a high prevalence of these methods so did not create distinct categories, but recorded details when they arose. *Other* was also used to classify studies that did not focus on any specific technique of artificial intelligence. For example, Sola et al. (2018) studied physician perceptions of

	artificial intelligence in general without isolating any particular artificial techniques.[89]
Reviewer who extracted the data	Initials of the person who (re)read the full text article and assigned values for the seven key characteristics outlined above: JKK, ALT, or DJL.
Reviewer notes	Optional free form notes from the person extracting the data.

Legend: AI: Artificial Intelligence, PC: Primary Care.

Notes: Subfields are ordered according to appearance in the results section of the manuscript. Cited examples are from studies captured by our scoping review.

References:

78. Andrisevic N, Ejaz K, Rios-Gutierrez F, Alba-Flores R, Nordehn G, Burns S. Detection of heart murmurs using wavelet analysis and artificial neural networks. *Journal of Biomechanical Engineering*. 2005;127(6):899-904. doi:10.1115/1.2049327

79. Safarova MS, Liu H, Kullo IJ. Rapid identification of familial hypercholesterolemia from electronic health records: The SEARCH study. *Journal of Clinical Lipidology*. 2016;10(5):1230-1239. doi:10.1016/j.jacl.2016.08.001

80. Kok MR, Boon ME, Schreiner-Kok PG, Hermans J, Grobbee DE, Kok LP. Less medical intervention after sharp demarcation of grade 1-2 cervical intraepithelial neoplasia smears by neural network screening. *Cancer*. 2001;93(3):173-178. doi:10.1002/cncr.9026

81. Teles G, Oliveira C, Braga R, et al. Using Bayesian networks to improve the decision-making process in public health systems. In: ; 2015:565-570. doi:10.1109/HealthCom.2014.7001904

82. Zouridakis G, Wadhawan T, Situ N, et al. Melanoma and other skin lesion detection using smart handheld devices. *Methods in molecular biology (Clifton, NJ)*. 2015;1256(bu3, 9214969):459-496. doi:10.1007/978-1-4939-2172-0_30

83. Soler JK, Corrigan D, Kazienko P, et al. Evidence-based rules from family practice to inform family practice; The learning healthcare system case study on urinary tract infections. *BMC Family Practice*. 2015;16(1). doi:10.1186/s12875-015-0271-4

84. Lange LL, Haak SW, Lincoln MJ, et al. Use of Iliad to Improve Diagnostic Performance of Nurse Practitioner Students. *Journal of Nursing Education*. 1997;36(1):36-45.

85. Katigari MR, Ayatollahi H, Malek M, Haghighi MK. Fuzzy expert system for diagnosing diabetic neuropathy. *World Journal of Diabetes*. 2017;8(2):80-88. doi:10.4239/wjd.v8.i2.80

86. Koeling R, Tate AR, Carroll JA. Automatically estimating the incidence of symptoms recorded in GP free text notes. In: ACM; 2011. doi:10.1145/2064747.2064757

87. Cox AP, Raluy-Callado M, Wang M, Bakheit AM, Moore AP, Dinet J. Predictive analysis for identifying potentially undiagnosed post-stroke spasticity patients in United Kingdom. *Journal of Biomedical Informatics*. 2016;60:328-333. doi:10.1016/j.jbi.2016.02.012

88. Newcomer SR, Steiner JF, Bayliss EA. Identifying Subgroups of Complex Patients With Cluster Analysis. *American Journal of Managed Care*. 2011;17(8):E324-E332.

89. Sola D, Borioli GS, Quaglia R. Predicting GPs' engagement with artificial intelligence. *British Journal of Health Care Management*. 2018;24(3):134-140. doi:10.12968/bjhc.2018.24.3.134

Table 2S: Complete author appointment counts.

Appointment Type	Number of studies with at least one author with the corresponding appointment
Biological and Medical Sciences	29
Company	49
Computer Science	97
Engineering	71
Epidemiology and Biostatistics	23
Health Sciences	33
Informatics	63
Mathematics	16
Medicine – Other	94
Medicine – Specialty	99
Medicine – Family or Primary Care	57
Nursing	4
Public Health	20
Statistics	15
Other	132
Unknown	110

Note: Each study fulfills one or more appointment type categories; each category is counted a maximum of one time for any given study.

Figure 2S Detailed Breakdown of Primary Care Functions

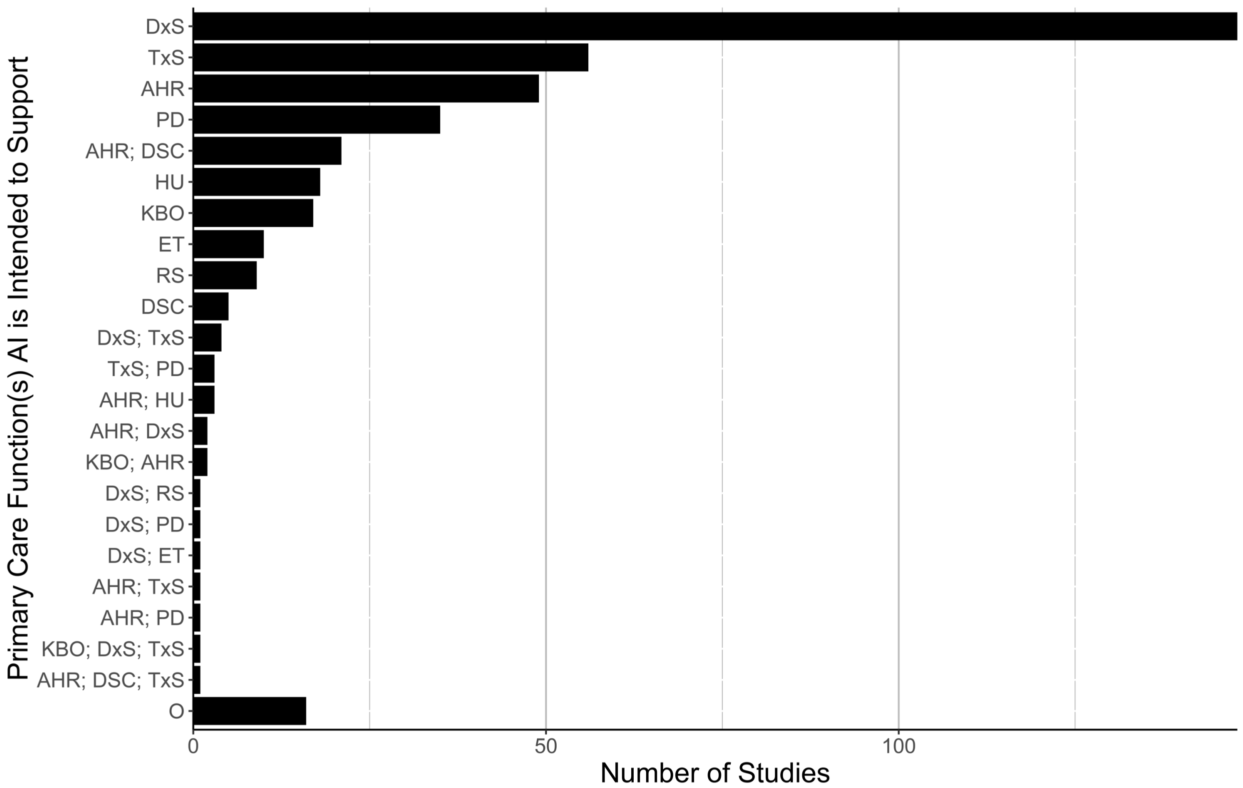

Legend: AHR: Analyze Health Records, DSC: Descriptives, ET: Education or Training, KBO: Knowledge Base or Ontology Construction, PD: Prediction, DxS: Diagnostic Decision Support, RS: Referral Support,TxS: Treatment Decision Support, HU: Health Care Use, O: Other

Figure 3S-A Author Reported End User Total Counts

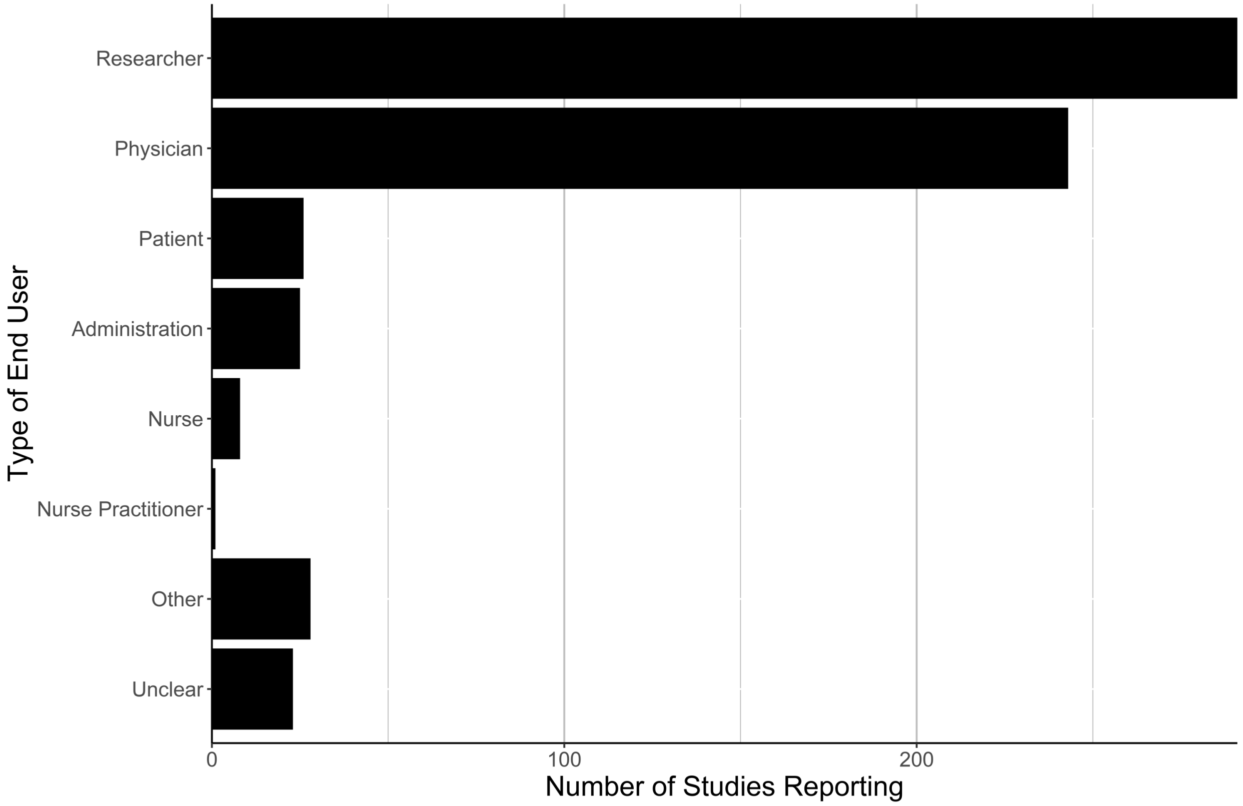

Note: A single study may contribute towards the count for one or more end user categories.

Figure 3S-B: Detailed Breakdown of Author Reported Intended End User Combinations by Study

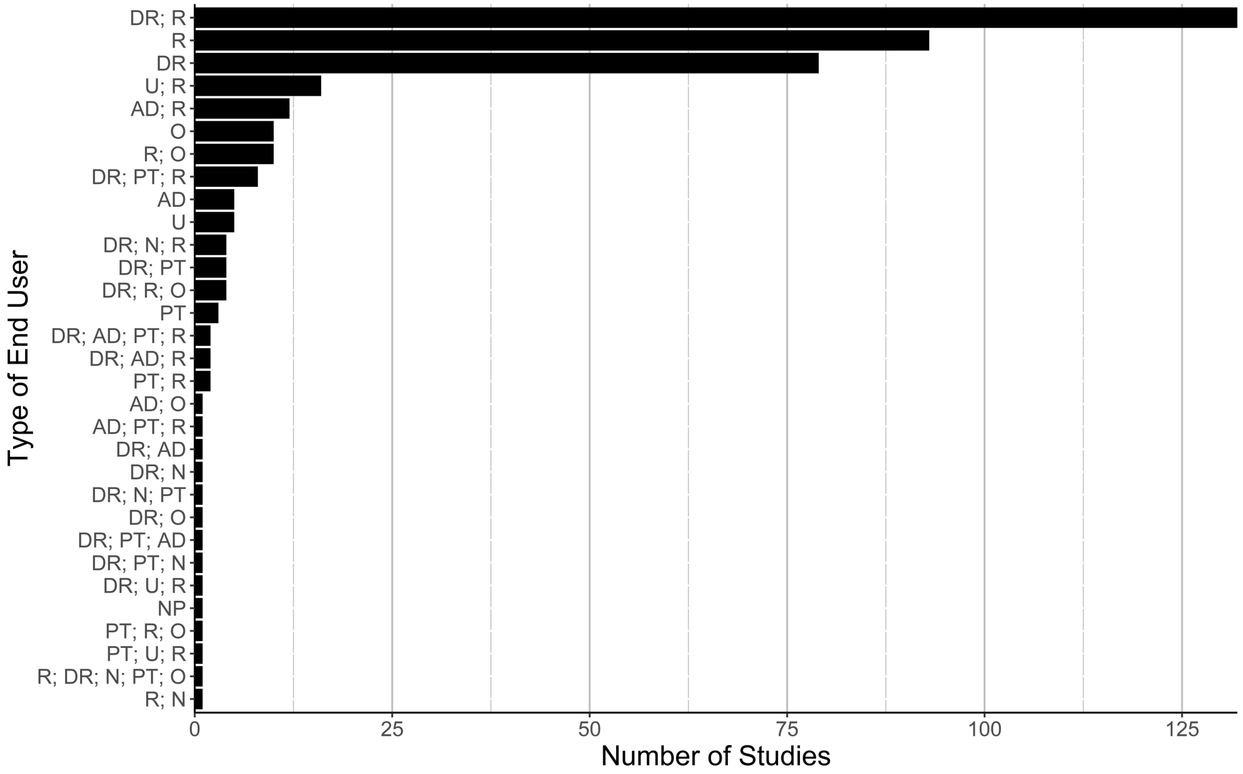

Legend: DR: Physician, R: Researcher, U: Unknown, AD: Administrator, PT: Patient, N: Nurse, NP: Nurse Practitioner, O: Other

Figure 4S Detailed Breakdown of Health Conditions

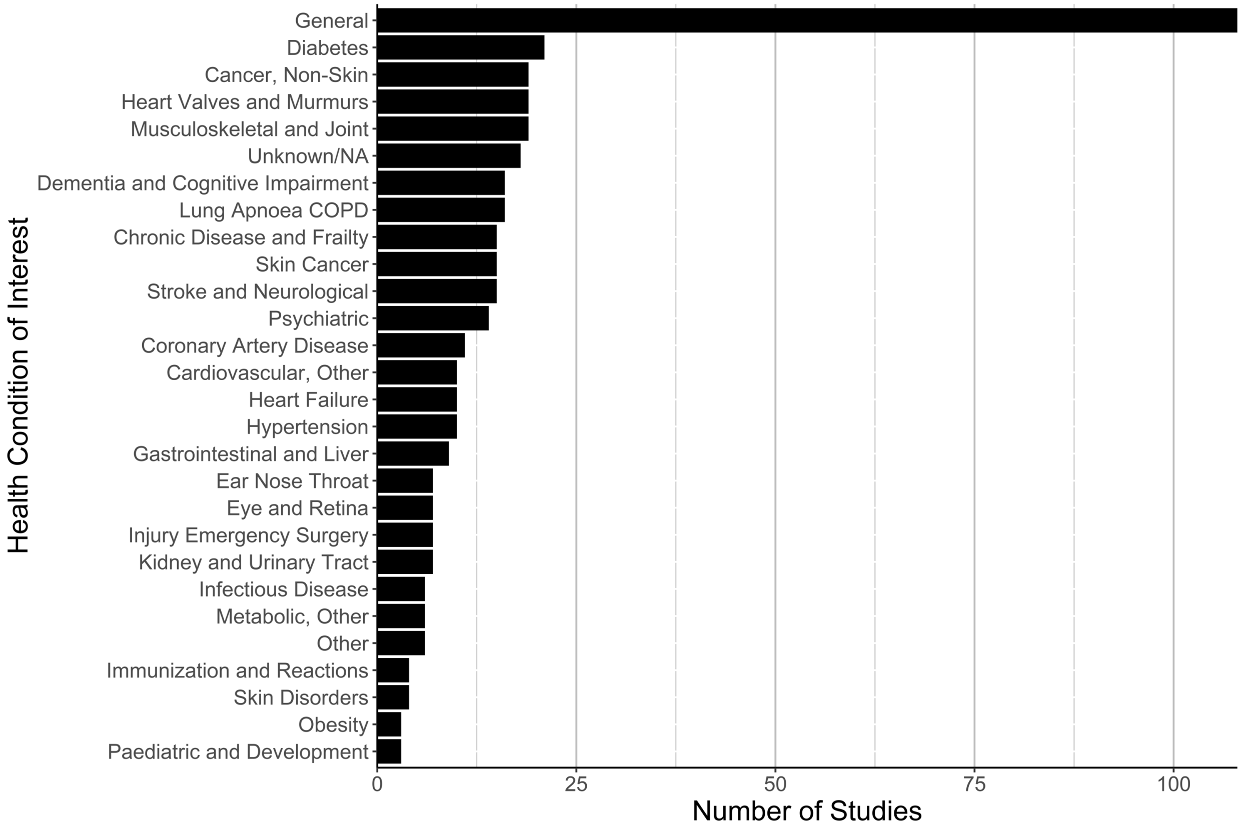

Figure 5S Most Frequent Locations of Data Source or Intended Implementation with Per Capita Rates

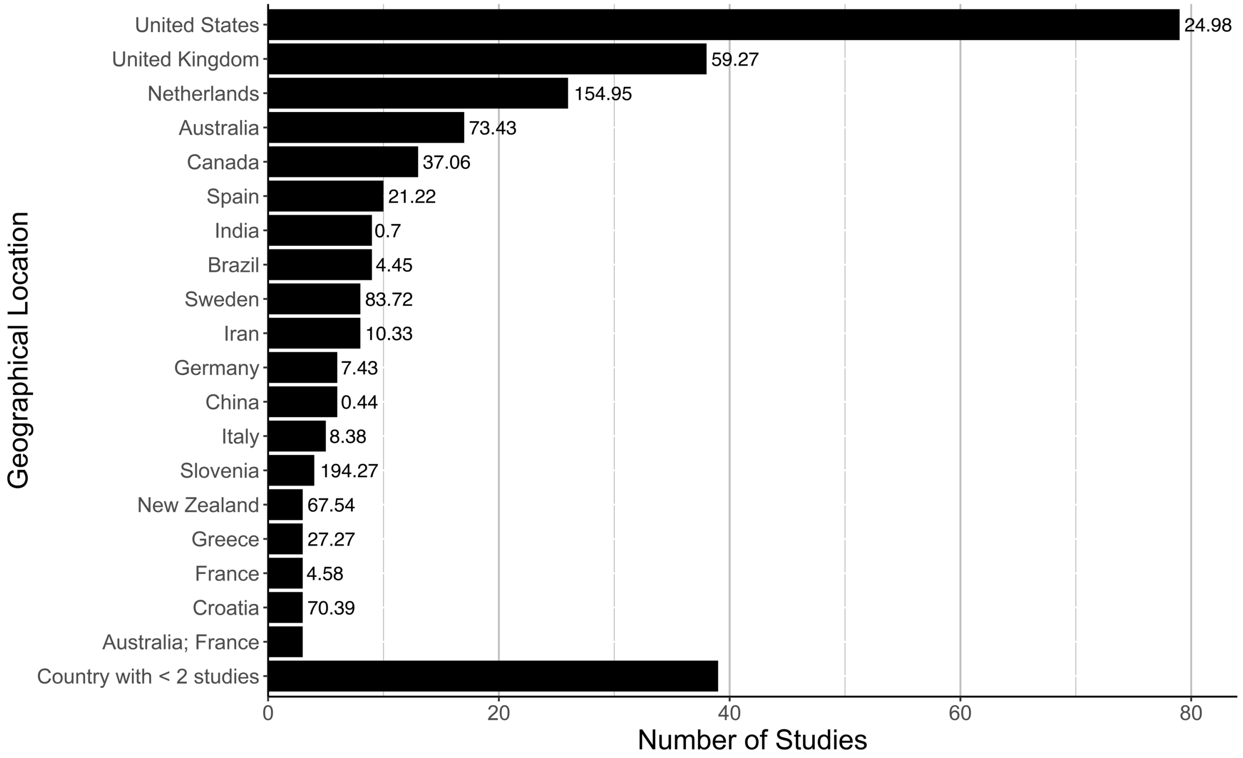

Notes: Only studies with location reported are included (n=292).
Number at the end of each bar is the number of studies per 100,000,000 people, based on 2013 population estimates.

Table 3S: Detailed breakdown of location

Location	Number of Studies
Unknown or Not Applicable	113
United States	79
United Kingdom	38
Netherlands	26
Australia	17
Canada	13
Spain	10
Brazil	9
India	9
Iran	8
Sweden	8
China	6
Germany	6
Italy	5
Slovenia	4
Australia and France	3

Croatia	3
France	3
Greece	3
New Zealand	3
Belgium	2
Egypt	2
Finland	2
Ireland	2
Japan	2
Norway	2
Singapore	2
Taiwan	2
Austria	1
Barcelona	1
Bulgaria	1
Canada and United States and United Kingdom and Brazil and Netherlands and Australia	1
Colombia	1
Czech Republic	1
Denmark	1
Europe	1
Germany and Norway	1
Greece and Bulgaria and Albania and Fyrom and Turkey	1
Hong Kong	1
Israel	1
Kuwait	1
Malaysia	1
Malta and Netherlands	1
Portugal	1
Saudi Arabia	1
South Africa	1
Switzerland	1
Turkey	1
United Kingdom and Greece and Germany	1
United States and Panama	1
United States and United Kingdom	1

Figure 6S-A Most Frequent Subfields of Artificial Intelligence with Median Year of Publication

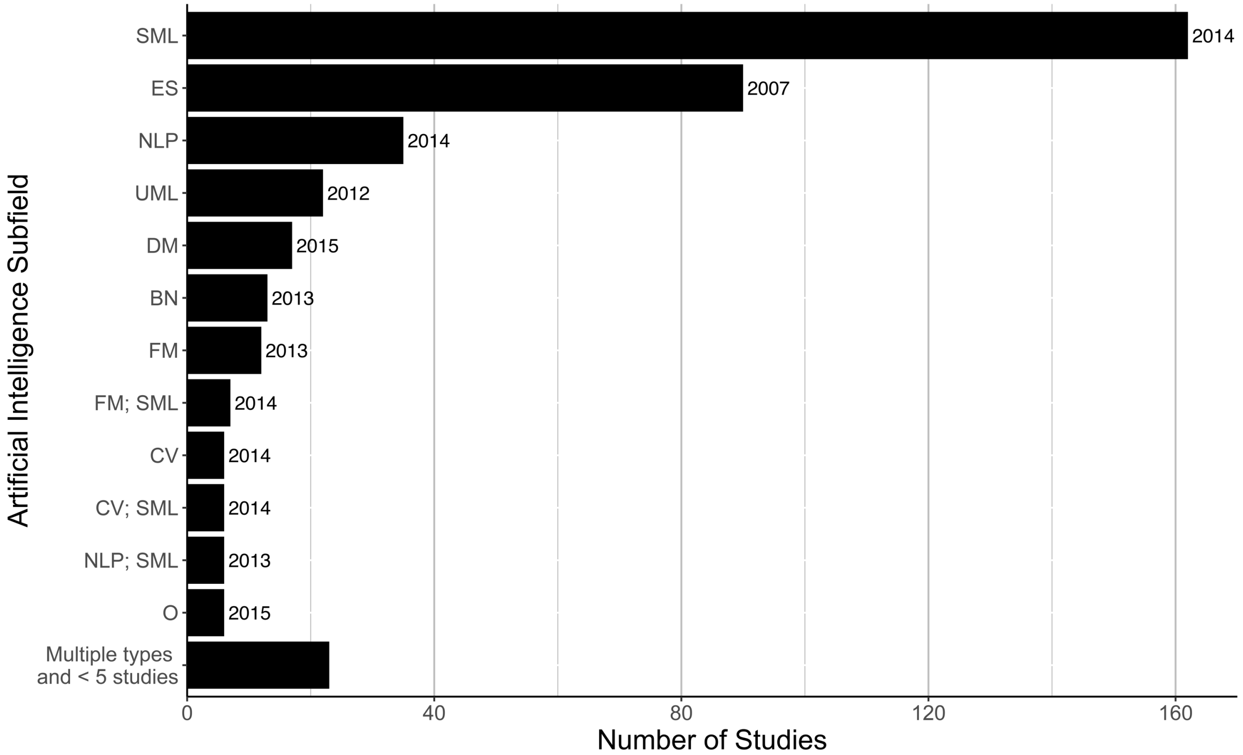

Legend: SML: Supervised Machine Learning, ES: Expert System, NLP: Natural Language Processing, UML: Unsupervised Machine Learning, DM: Data Mining, BN: Bayesian Network, FM: Fuzzy Models, O: Other

Figure 6S-B: Detailed Breakdown of Artificial Intelligence Subfields with Median Year of Publication

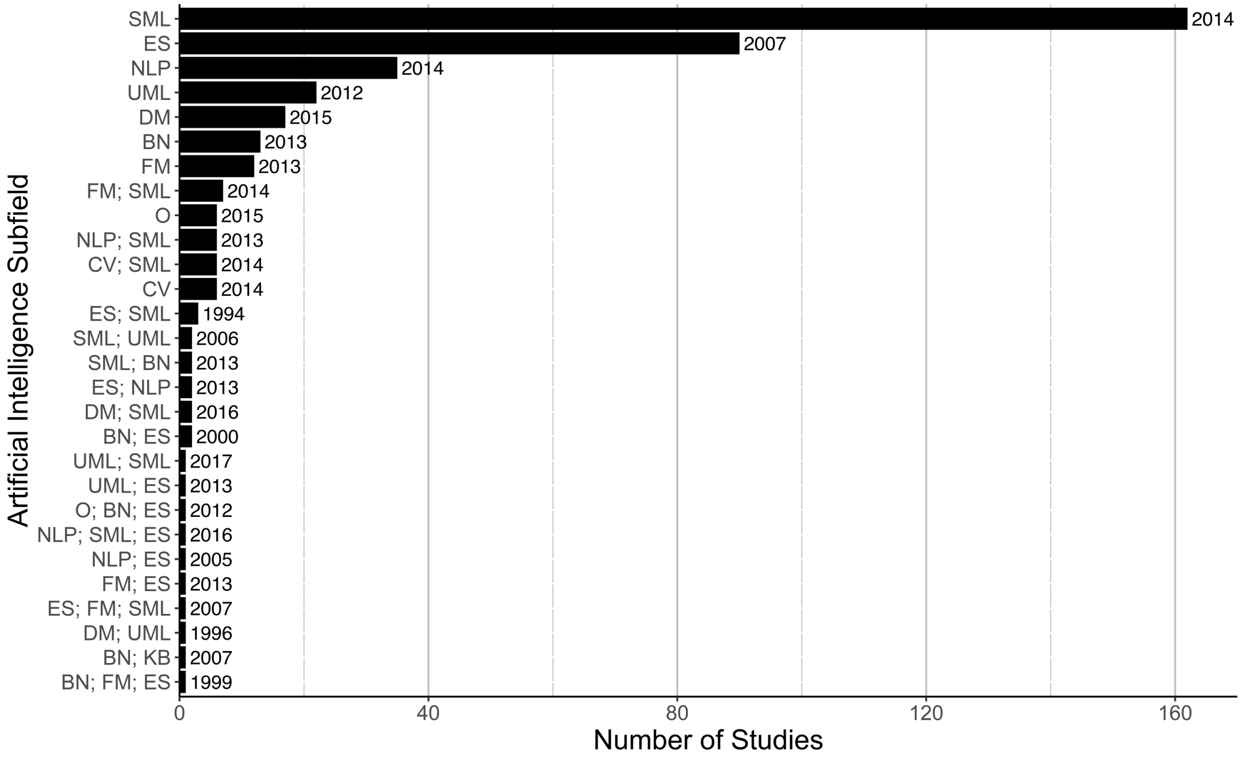

Legend: SML: Supervised Machine Learning, ES: Expert System, NLP: Natural Language Processing, UML: Unsupervised Machine Learning, DM: Data Mining, BN: Bayesian Network, FM: Fuzzy Models, O: Other

SUPPLEMENTAL APPENDIX 4: REFERENCES

1. Abdel-Aal RE, Mangoud AM. Modeling obesity using abductive networks. *Computers and Biomedical Research*. 1997;30(6):451-471. doi:10.1006/cbmr.1997.1460

2. Abe K, Takagi H, Minami M, Tian H. Features for measuring the congestive extent of internal hemorrhoids in endoscopic images. In: *Proceedings of the Eleventh Australasian Data Mining Conference*. Vol 146. Canberra, Australia: Conferences in Research and Practice in Information Technology Series; 2013:8.

3. Abidi S, Cox J, Shepherd M, Abidi SSR. Using OWL ontologies for clinical guidelines based comorbid decision support. In: Maui, HI, USA: IEEE Computer Society; 2012:3030-3038. doi:10.1109/HICSS.2012.629

4. Abidi SR, Abidi SS, Hussain S, Shepherd M. Ontology-based modeling of clinical practice guidelines: a clinical decision support system for breast cancer follow-up interventions at primary care settings. *MEDINFO*. 2007:845-849.

5. Abidi SR. Ontology-based modeling of breast cancer follow-up clinical practice guideline for providing clinical decision support. In: *Twentieth IEEE International Symposium on Computer-Based Medical Systems (CBMS'07)*. Maribor, Slovenia: IEEE; 2007:542-547. doi:10.1109/CBMS.2007.80

6. Adjah ESO, Montvida O, Agbeve J, Paul SK. Data mining approach to identify disease cohorts from primary care electronic medical records: a case of diabetes mellitus. *The Open Bioinformatics Journal*. 2017;10(1):16-27. doi:10.2174/1875036201710010016

7. Aerts M, Minalu G, Bösner S, et al. Pooled individual patient data from five countries were used to derive a clinical prediction rule for coronary artery disease in primary care. *Journal of Clinical Epidemiology*. 2017;81:120-128. doi:10.1016/j.jclinepi.2016.09.011

8. af Klercker T, Zetraeus S. Dilemmas in introducing World Wide Web-based information technology in primary care: a focus group study. *Family Practice*. 1988;15(3):205-210.

9. af Klercker T. Effects of pruning of a decision-tree for the ear, nose, and throat realm in primary health care based on case-notes. *Journal of Medical Systems*. 1996;20(4):215-226. doi:10.1007/BF02263393

10. Afifi S, GholamHosseini H, Sinha R. A low-cost FPGA-based SVM classifier for melanoma detection. In: Malaysia: IEEE; 2016:631-636. doi:10.1109/IECBES.2016.7843526

11. Afifi S, GholamHosseini H, Sinha R. SVM classifier on chip for melanoma detection. In: Seogwipo: IEEE; 2017:270-274. doi:10.1109/EMBC.2017.8036814

12. Afzal Z, Kors JA, Sturkenboom MC, Schuemie MJ. Identifying drug-safety signals in electronic health records: An evaluation of automated case-detection algorithms with different

sensitivity and specificity. *Pharmacoepidemiology and Drug Safety*. 2013;22(s1):285. doi:10.1002/pds.3512
13. Afzal Z, Schuemie MJ, Sen E, Jong G, Sturkenboom MC, Kors JA. Automatic generation of a case-detection algorithm for hepatobiliary disease using machine learning on free-text electronic health records. In: Vol 21. Pharmacoepidemiology and Drug Safety; 2012:194.

14. Afzal Z, Engelkes M, Verhamme KMC, et al. Automatic generation of case-detection algorithms to identify children with asthma from large electronic health record databases: automated case-detection algorithms. *Pharmacoepidemiology and Drug Safety*. 2013;22(8):826-833. doi:10.1002/pds.3438

15. Afzal Z, Pons E, Kang N, Sturkenboom MC, Schuemie MJ, Kors JA. ContextD: an algorithm to identify contextual properties of medical terms in a Dutch clinical corpus. *BMC Bioinformatics*. 2014;15(1). doi:10.1186/s12859-014-0373-3

16. Ahlstrom C, Hult P, Rask P, et al. Feature extraction for systolic heart murmur classification. *Annals of Biomedical Engineering*. 2006;34(11):1666-1677. doi:10.1007/s10439-006-9187-4

17. Al-Hamadani B. CardioOWL: An ontology-driven expert system for diagnosing coronary artery diseases. In: Subang, Selangor, Malaysia: IEEE; 2014:128-132. doi:10.1109/ICOS.2014.7042642

18. Alendahl K, Timpka T, Sjoberg C. Computerized knowledge bases in primary health care- a curse or a blessing for health promotion, prevention and patient quality. *MEDINFO*. 1995;95:917-921.

19. Alhashmi SM. Design of an internet-based advisory system: a multi-agent approach. In: Bui TD, Ho TV, Ha QT, eds. *Intelligent Agents and Multi-Agent Systems*. Vol 5357. Berlin, Heidelberg: Springer Berlin Heidelberg; 2008:14-25. doi:10.1007/978-3-540-89674-6_5

20. Ali Z, Alsulaiman M, Muhammad G, et al. Intra- and inter-database study for Arabic, English, and German databases: do conventional speech features detect voice pathology? *Journal of Voice*. 2017;31(3):386.e1-386.e8. doi:10.1016/j.jvoice.2016.09.009

21. Almansoori W, Zarour O, Jarada TN, Karampales P, Rokne J, Alhajj R. Applications of social network construction and analysis in the medical referral process. In: *2011 IEEE 9th International Conference on Dependable, Autonomic and Secure Computing*. Sydney, Australia: IEEE; 2011:816-823. doi:10.1109/DASC.2011.140

22. Alty SR, Angarita-Jaimes N, Millasseau SC, Chowienczyk PJ. Predicting arterial stiffness from the digital volume pulse waveform. *IEEE Transactions on Biomedical Engineering*. 2007;54(12):2268-2275. doi:10.1109/TBME.2007.897805

23. Alvarez-Guisasola F, Conget I, Franch J, et al. Adding questions about cardiovascular risk factors improve the ability of the ADA questionnaire to identify unknown diabetic patients in Spain. *Av Diabetol*. 2010;26:347-352.

24. Alves R, Piñol M, Vilaplana J, et al. Computer-assisted initial diagnosis of rare diseases. *PeerJ*. 2016;4:e2211. doi:10.7717/peerj.2211

25. Anchala R, Di Angelantonio E, Prabhakaran D, Franco OH. Development and validation of a clinical and computerised decision support system for management of hypertension (DSS-HTN) at a primary health care (PHC) setting. Magrabi F, ed. *PLoS ONE*. 2013;8(11):e79638. doi:10.1371/journal.pone.0079638

26. Andrisevic N, Ejaz K, Rios-Gutierrez F, Alba-Flores R, Nordehn G, Burns S. Detection of heart murmurs using wavelet analysis and artificial neural networks. *Journal of Biomechanical Engineering*. 2005;127(6):899. doi:10.1115/1.2049327

27. Anwer A, Prasinos M, Bamiou D-E, et al. EMBalance data repository modeling and clinical application. In: *IEEE 15th International Conference on Bioinformatics and Bioengineering*. Belgrade, Serbia: IEEE; 2015. https://ieeexplore.ieee.org/document/7367677.

28. Aref-Eshghi E, Oake J, Godwin M, et al. Identification of dyslipidemic patients attending primary care clinics using electronic medical record (EMR) data from the Canadian Primary Care Sentinel Surveillance Network (CPCSSN) Database. *Journal of Medical Systems*. 2017;41(3). doi:10.1007/s10916-017-0694-7

29. Arnold CW, Oh A, Chen S, Speier W. Evaluating topic model interpretability from a primary care physician perspective. *Computer Methods and Programs in Biomedicine*. 2016;124:67-75. doi:10.1016/j.cmpb.2015.10.014

30. Astilean A, Avram C, Folea S, Silvasan I, Petreus D. Fuzzy Petri nets based decision support system for ambulatory treatment of non-severe acute diseases. In: *2010 IEEE International Conference on Automation, Quality and Testing, Robotics (AQTR)*. Cluj-Napoca, Romania: IEEE; 2010:1-6. doi:10.1109/AQTR.2010.5520706

31. Avila ML, Brandao LR, Wiliams S, et al. A simple pediatric post-thrombotic syndrome classification algorithm for the general practitioner. In: Vol 13. 2015:220.

32. Babic F, Vadovsky M, Muchova M, Paralic J, Majnaric L. Simple understandable analysis of medical data to support the diagnostic process. In: *2017 IEEE 15th International Symposium on Applied Machine Intelligence and Informatics (SAMI)*. Herl'any, Slovakia: IEEE; 2017:000153-000158. doi:10.1109/SAMI.2017.7880293

33. Baez GP, Viadero CF, Perez del Pino MA, Prochazka A, Suarez Araujo CP. HUMANN-based systems for differential diagnosis of dementia using neuropsychological tests. In: *2010 IEEE 14th International Conference on Intelligent Engineering Systems*. Las Palmas: IEEE; 2010:67-72. doi:10.1109/INES.2010.5483872

34. Baez PG, Viadero CF, Espinosa NR, Perez del Pino MA, Suarez-Araujo CP. Detection of mild cognitive impairment using a counter propagation network based system. An e-health

solution. In: *2015 International Workshop on Computational Intelligence for Multimedia Understanding (IWCIM)*. Prague, Czech Republic: IEEE; 2015:1-5. doi:10.1109/IWCIM.2015.7347094

35. Báez PG, Araujo CPS, Viadero CF, Procházka A. Differential diagnosis of dementia using HUMANN-S based ensembles. In: Fodor J, Klempous R, Suárez Araujo CP, eds. *Recent Advances in Intelligent Engineering Systems*. Vol 378. Berlin, Heidelberg: Springer Berlin Heidelberg; 2012:305-324. doi:10.1007/978-3-642-23229-9_14

36. Báez P, Viadero CF, García JR, Araujo CPS. An ensemble approach for the diagnosis of cognitive decline with missing data. In: Corchado E, Abraham A, Pedrycz W, eds. *Hybrid Artificial Intelligence Systems*. Vol 5271. Berlin, Heidelberg: Springer Berlin Heidelberg; 2008:353-360. doi:10.1007/978-3-540-87656-4_44

37. Balas EA, Li ZR, Spencer DC, Jaffrey F, Brent E, Mitchell JA. An expert system for performance-based direct delivery of published clinical evidence. *Journal of the American Medical Informatics Association*. 1996;3(1):56-65. doi:10.1136/jamia.1996.96342649

38. Barons MJ, Parsons N, Griffiths F, Thorogood M. A comparison of artificial neural network, latent class analysis and logistic regression for determining which patients benefit from a cognitive behavioural approach to treatment for non-specific low back pain. In: *2013 IEEE Symposium on Computational Intelligence in Healthcare and E-Health (CICARE)*. Singapore, Singapore: IEEE; 2013:7-12. doi:10.1109/CICARE.2013.6583061

39. Bekker J, Hommersom A, Lappenschaar M, Davis J. Measuring adverse drug effects on multimorbidity using tractable bayesian networks. 2016. http://arxiv.org/abs/1612.03055. Accessed July 3, 2019.

40. Beliakov G, Warren J. Fuzzy logic for decision support in chronic care. *Artificial Intelligence in Medicine*. 2001;21(1-3):209-213. doi:10.1016/S0933-3657(00)00087-7

41. Bermejo P, Lucas M, Rodríguez-Montes JA, et al. Single- and multi-label prediction of burden on families of schizophrenia patients. In: Peek N, Marín Morales R, Peleg M, eds. *Artificial Intelligence in Medicine*. Vol 7885. Berlin, Heidelberg: Springer Berlin Heidelberg; 2013:115-124. doi:10.1007/978-3-642-38326-7_18

42. Beveridge M, Fox J. Automatic generation of spoken dialogue from medical plans and ontologies. *Journal of Biomedical Informatics*. 2006;39(5):482-499. doi:10.1016/j.jbi.2005.12.008

43. Bhattacharya A, Mishra M, Singh A, Dutta MK. Machine learning based portable device for detection of cardiac abnormality. In: *2017 International Conference on Emerging Trends in Computing and Communication Technologies (ICETCCT)*. Dehradun: IEEE; 2017:1-4. doi:10.1109/ICETCCT.2017.8280307

44. Biermann E, Rihl J, Schenker M, Standl E. Semi-automatic generation of medical tele-expert opinion for primary care physician. *Methods of Information in Medicine*. 2003;42(03):212-219. doi:10.1055/s-0038-1634353

45. Biermans MCJ, Verheij RA, de Bakker DH, Zielhuis GA, de Vries Robbé PF. Estimating morbidity rates from electronic medical records in general practice: evaluation of a grouping system. *Methods of Information in Medicine*. 2008;47(02):98-106. doi:10.3414/ME0471

46. Biermans MCJ, de Bakker DH, Verheij RA, Gravestein JV, van der Linden MW, de Vries Robbé PF. Development of a case-based system for grouping diagnoses in general practice. *International Journal of Medical Informatics*. 2008;77(7):431-439. doi:10.1016/j.ijmedinf.2007.08.002

47. Bindels R, de Clercq PA, Winkens RAG, Hasman A. A test ordering system with automated reminders for primary care based on practice guidelines. *International Journal of Medical Informatics*. 2000;58-59:219-233. doi:10.1016/S1386-5056(00)00089-7

48. Bindels R, Hasman A, van Wersch JWJ, Talmon J, Winkens RAG. Evaluation of an automated test ordering and feedback system for general practitioners in daily practice. *International Journal of Medical Informatics*. 2004;73(9-10):705-712. doi:10.1016/j.ijmedinf.2004.06.001

49. Bland JDP, Weller P, Rudolfer S. Questionnaire tools for the diagnosis of carpal tunnel syndrome from the patient history. *Muscle & Nerve*. 2011;44(5):757-762. doi:10.1002/mus.22158

50. Boilot P, Hinesa E, John S, et al. Detection of bacteria causing eye infections using a neural network based electronic nose system. In: *Section 4: Medical/Microbial*. Electronic Noses and Olfaction; 2000:8.

51. Bourdès V, Ferrières J, Amar J, et al. Prediction of persistence of combined evidence-based cardiovascular medications in patients with acute coronary syndrome after hospital discharge using neural networks. *Medical & Biological Engineering & Computing*. 2011;49(8):947-955. doi:10.1007/s11517-011-0785-4

52. Brennan PF, Aronson AR. Towards linking patients and clinical information: detecting UMLS concepts in e-mail. *Journal of Biomedical Informatics*. 2003;36(4-5):334-341. doi:10.1016/j.jbi.2003.09.017

53. Bulegon H, Bortoleto S. Developing through a modeling of the database: an implementation of PEP for cardiovascular monitoring. In: *World Congress on Nature and Biologically Inspired Computing*. IEEE; 2009.

54. Bulegon H, Bortoleto S, Roman AC. Developing an implementation through a modeling of the database for cardiovascular monitoring. In: *2009 Ninth International Conference on*

Intelligent Systems Design and Applications. Pisa, Italy: IEEE; 2009:1156-1160. doi:10.1109/ISDA.2009.129

55. Buntinx F, Truyen J, Embrechts P, Moreel G, Peeters R. Evaluating patients with chest pain using classification and regression trees. *Family Practice*. 1992;9(2):149-153. doi:10.1093/fampra/9.2.149

56. Butler H, Baker M, Hegarty M, Finlayson D. A spectroscopic serum based blood test for brain tumours: optimisation for high-throughput sampling and the health economic impacts. In: Neuro-Oncology; 2018:i4.

57. Byrd RJ, Steinhubl SR, Sun J, Ebadollahi S, Stewart WF. Automatic identification of heart failure diagnostic criteria, using text analysis of clinical notes from electronic health records. *International Journal of Medical Informatics*. 2014;83(12):983-992. doi:10.1016/j.ijmedinf.2012.12.005

58. Cardillo E, Chiaravalloti MT, Pasceri E. Assessing ICD-9-CM and ICPC-2 use in primary care. An Italian case study. In: *Proceedings of the 5th International Conference on Digital Health 2015 - DH '15*. Florence, Italy: ACM Press; 2015:95-102. doi:10.1145/2750511.2750525

59. Cardillo E, Eccher C, Serafini L, Tamilin A. Logical analysis of mappings between medical classification systems. In: Dochev D, Pistore M, Traverso P, eds. *Artificial Intelligence: Methodology, Systems, and Applications*. Vol 5253. Berlin, Heidelberg: Springer Berlin Heidelberg; 2008:311-321. doi:10.1007/978-3-540-85776-1_26

60. Cardillo E, Serafini L, Tamilin A. A hybrid methodology for consumer-oriented healthcare knowledge acquisition. In: Springer-Verlag Berlin Heidelberg; 2010:38-49.

61. Carrara M, Bono A, Bartoli C, et al. Multispectral imaging and artificial neural network: mimicking the management decision of the clinician facing pigmented skin lesions. *Physics in Medicine and Biology*. 2007;52(9):2599-2613. doi:10.1088/0031-9155/52/9/018

62. Chamberlain D, Kodgule R, Ganelin D, Miglani V, Fletcher RR. Application of semi-supervised deep learning to lung sound analysis. In: *2016 38th Annual International Conference of the IEEE Engineering in Medicine and Biology Society (EMBC)*. Orlando, FL, USA: IEEE; 2016:804-807. doi:10.1109/EMBC.2016.7590823

63. Chande PK, Katiyal S, Shrivastava M, Ramani AK. Expert based uroflow metering system. In: *Proceedings IECON '91: 1991 International Conference on Industrial Electronics, Control and Instrumentation*. Kobe, Japan: IEEE; 1991:1521-1524. doi:10.1109/IECON.1991.239110

64. Chang W-Y, Huang A, Chen Y-C, et al. The feasibility of using manual segmentation in a multifeature computer-aided diagnosis system for classification of skin lesions: a retrospective comparative study. *BMJ Open*. 2015;5(4):e007823-e007823. doi:10.1136/bmjopen-2015-007823

65. Chao J, Gu J, Zhang H, Chen H, Wu Z. The impact of the National Essential Medicines Policy on rational drug use in primary care institutions in Jiangsu Province of China. *Iran J Public Health*. 2018;47(1):24-32.

66. Chase HS, Mitrani LR, Lu GG, Fulgieri DJ. Early recognition of multiple sclerosis using natural language processing of the electronic health record. *BMC Medical Informatics and Decision Making*. 2017;17(1). doi:10.1186/s12911-017-0418-4

67. Chen J, Zheng X. A system architecture for smart health services and applications. In: Bikakis A, Zheng X, eds. *Multi-Disciplinary Trends in Artificial Intelligence*. Vol 9426. Cham: Springer International Publishing; 2015:449-456. doi:10.1007/978-3-319-26181-2_42

68. Chen J, Druhl E, Polepalli Ramesh B, et al. A natural language processing system that links medical terms in electronic health record notes to lay definitions: system development using physician reviews. *Journal of Medical Internet Research*. 2018;20(1):e26. doi:10.2196/jmir.8669

69. Chen S, Gutkowicz-Krusin D, Adrian J, Wells R. Performance of an adjuvant melanoma detection tool compared to physicians. In: Vol 64. J Am Acad Dermatol; 2011:AB121.

70. Choi E, Schuetz A, Stewart WF, Sun J. Using recurrent neural network models for early detection of heart failure onset. *AMIA*. 2017;24(2):361-370. doi:10.1093/jamia/ocw112

71. Chowdhury S, Linnarsson R, Wallgren A, Wallgren B, Wigertz O. Extracting knowledge from a large primary health care database using a knowledge-based statistical approach. *Journal of Medical Systems*. 1990;14(4):213-225. doi:10.1007/BF00999270

72. Çınar M, Engin M, Engin EZ, Ziya Ateşçi Y. Early prostate cancer diagnosis by using artificial neural networks and support vector machines. *Expert Systems with Applications*. 2009;36(3):6357-6361. doi:10.1016/j.eswa.2008.08.010

73. Closs VE, Ziegelmann PK, Flores JHF, Gomes I, Schwanke CHA. Anthropometric measures and frailty prediction in the elderly: an easy-to-use tool. *Current Gerontology and Geriatrics Research*. 2017;2017:1-8. doi:10.1155/2017/8703503

74. Cox A, Dinet J, Raluy-Callado M, MacLachlan S, Gabriel S. Resource utilisation and costs in patients with post-stroke spasticity in the United Kingdom. In: Vol 31. London: Clinical Rehabilitation; 2015:849. doi:10.1177/0269215517698029

75. Cox AP, Raluy-Callado M, Wang M, Bakheit AM, Moore AP, Dinet J. Predictive analysis for identifying potentially undiagnosed post-stroke spasticity patients in United Kingdom. *Journal of Biomedical Informatics*. 2016;60:328-333. doi:10.1016/j.jbi.2016.02.012

76. Cruz-Gutiérrez V, Posada-Zamora MA, Sánchez-López A. An Efficient Expert System for Diabetes with a Bayesian Inference Engine. In: Pichardo-Lagunas O, Miranda-Jiménez S, eds. *Advances in Soft Computing*. Vol 10062. Cham: Springer International Publishing; 2017:54-64. doi:10.1007/978-3-319-62428-0_5

77. Cubillas JJ, Ramos MI, Feito FR, Ureña T. An improvement in the appointment scheduling in primary health care centers using data mining. *Journal of Medical Systems*. 2014;38(8). doi:10.1007/s10916-014-0089-y

78. Das S, Sil J. Uncertainty management of health attributes for primary diagnosis. In: *2017 International Conference on Big Data Analytics and Computational Intelligence (ICBDAC)*. Chirala, Andhra Pradesh, India: IEEE; 2017:360-365. doi:10.1109/ICBDACI.2017.8070864

79. Dawson G, Dourish C, Goodwin G, et al. A precision medicine approach to antidepressant treatment in depression. In: Vol 40. Neuropsychopharmacology; 2015:S176. doi:10.1038/npp.2015.325

80. Dawson GR, Kingslake J., Dourish C.T., et al. A precision medicine approach to antidepressant treatment in depression. In: *Assuring Replication: Problems and Solutions*. Vol 79. Biological Psychiatry; 2016:S215. doi:10.1016/j.biopsych.2016.03.1054

81. de Groot H, Lous J, Peters L, Sandell A, Onell A. An allergy decision support system for non-allergy specialists: test and evaluation in routine use. In: *New Findings in Allergy Diagnosis*. Vol 64. Blackwell Publishing Ltd; 2009:533.

82. de Ipiña KL, Hernández MC, Graña M, Martínez E, Vaquero C. A computer-aided decision support system for shoulder pain pathology. In: Demazeau Y, Dignum F, Corchado JM, et al., eds. *Trends in Practical Applications of Agents and Multiagent Systems*. Vol 71. Berlin, Heidelberg: Springer Berlin Heidelberg; 2010:705-712. doi:10.1007/978-3-642-12433-4_83

83. de Ipiña KL, Hernández MC, Martínez E, Vaquero C. On the design of a CADS for shoulder pain pathology. In: Graña Romay M, Corchado E, Garcia Sebastian MT, eds. *Hybrid Artificial Intelligence Systems*. Vol 6076. Berlin, Heidelberg: Springer Berlin Heidelberg; 2010:508-515. doi:10.1007/978-3-642-13769-3_62

84. Denny JC, Peterson JF, Choma NN, et al. Development of a natural language processing system to identify timing and status of colonoscopy testing in electronic medical records. *AMIA*. 2009:141.

85. Denny JC, Choma NN, Peterson JF, et al. Natural language processing improves identification of colorectal cancer testing in the electronic medical record. *Medical Decision Making*. 2012;32(1):188-197. doi:10.1177/0272989X11400418

86. Dinet J, Cox AP, Raluy-Callado M, et al. Predictive analysis for identifying post stroke spasticity patients in UK primary care data. In: Vol 23. John Wiley & Sons, Ltd.; 2014:422. doi:10.1002/pds

87. Dixon BE, Simonaitis L, Goldberg HS, et al. A pilot study of distributed knowledge management and clinical decision support in the cloud. *Artificial Intelligence in Medicine*. 2013;59(1):45-53. doi:10.1016/j.artmed.2013.03.004

88. Dominguez-Morales JP, Jimenez-Fernandez AF, Dominguez-Morales MJ, Jimenez-Moreno G. Deep neural networks for the recognition and classification of heart murmurs using neuromorphic auditory sensors. *IEEE Trans Biomed Circuits Syst*. 2018;12(1):24-34. doi:10.1109/TBCAS.2017.2751545

89. Dowell A, Darlow B, Macrae J, Stubbe M, Turner N, McBain L. Childhood respiratory illness presentation and service utilisation in primary care: a six-year cohort study in Wellington, New Zealand, using natural language processing (NLP) software. *BMJ Open*. 2017;7(7):e017146. doi:10.1136/bmjopen-2017-017146

90. Downs SM, Wallace MY. Mining association rules from a pediatric primary care decision support system. *AMIA*. 2000:200-204.

91. Dravenstott R, Kirchner HL, Strömblad C, Boris D, Leader J, Devapriya P. Applying predictive modeling to identify patients at risk to no-show. In: *Proceedings of the 2014 Industrial and Systems Engineering Research Conference*. ; :10.

92. Duarte PS, Mastrocolla LE, Farsky PS, et al. Selection of patients for myocardial perfusion scintigraphy based on fuzzy sets theory applied to clinical-epidemiological data and treadmill test results. *Brazilian Journal of Medical and Biological Research*. 2006;39(1):9-18. doi:10.1590/S0100-879X2006000100002

93. Dubey AK. Using rough sets, neural networks, and logistic regression to predict compliance with cholesterol guidelines goals in patients with coronary artery disease. *AMIA*. 2003:834.

94. Dupuits FMHM. The use of the arden syntax for MLMs in HIOS +, a decision support system for general practitioners in the Netherlands. *Comput Biol Med*. 1994;24(5):405-410.

95. Dupuits FMHM. Evaluation of GPs' medical knowledge by a decision support system. *Computer methods and programs in biomedicine*. 1995;48:91-96.

96. Dupuits FMHM, Hasman A, Pop P. Computer-based assistance in family medicine. *Computer Methods and Programs in Biomedicine*. 1998;55(1):39-50. doi:10.1016/S0169-2607(97)00046-1

97. Ehrensperger MM, Taylor KI, Berres M, et al. BrainCheck - a very brief tool to detect incipient cognitive decline: optimized case-finding combining patient- and informant-based data. *Alzheimer's Research & Therapy*. 2014;6(9). doi:10.1186/s13195-014-0069-y

98. Eichler T, Thyrian JR, Fredrich D, et al. The benefits of implementing a computerized Intervention-Management-System (IMS) on delivering integrated dementia care in the primary care setting. *International Psychogeriatrics*. 2014;26(8):1377-1385. doi:10.1017/S1041610214000830

99. Emir B, Mardekian J, Masters ET, Clair A, Kuhn M, Silverman SL. Predictive modeling of a Fibromyalgia diagnosis: increasing the accuracy using real world data. In: *Fibromyalgia, Soft Tissue Disorders, Regional and Specific Clinical Pain Syndromes: Research Focus*. 2014:3.

100. Engelkes M, Afzal Z, Janssens HM, et al. Automated identification of asthma patients within an electronical medical record database using machine learning. In: *Pharmacoepidemiology and Drug Safety*. Vol 21. Wiley Online Library; 2012:12.

101. Ethier J-F, Curcin V, Barton A, et al. Clinical data integration model: core interoperability ontology for research using primary care data. *Methods of Information in Medicine*. 2015;54(01):16-23. doi:10.3414/ME13-02-0024

102. Exarchos TP, Rigas G, Bibas A, et al. Mining balance disorders' data for the development of diagnostic decision support systems. *Computers in Biology and Medicine*. 2016;77:240-248. doi:10.1016/j.compbiomed.2016.08.016

103. Fadhil A, Gabrielli S. Addressing challenges in promoting healthy lifestyles: the al-chatbot approach. In: Barcelona, Spain: Association for Computing Machinery; 2017.

104. Farmer N. An update and further testing of a knowledge-based diagnostic clinical decision support system for musculoskeletal disorders of the shoulder for use in a primary care setting: Musculoskeletal diagnostic CDSS. *Journal of Evaluation in Clinical Practice*. 2014;20(5):589-595. doi:10.1111/jep.12153

105. Farran B, Channanath AM, Behbehani K, Thanaraj TA. Predictive models to assess risk of type 2 diabetes, hypertension and comorbidity: machine-learning algorithms and validation using national health data from Kuwait—a cohort study. *BMJ Open*. 2013;3(5):e002457. doi:10.1136/bmjopen-2012-002457

106. Fathima SA, Hundewale N. Comparitive analysis of machine learning techniques for classification of arbovirus. In: *Proceedings of 2012 IEEE-EMBS International Conference on Biomedical and Health Informatics*. Hong Kong: IEEE; 2012:376-379. doi:10.1109/BHI.2012.6211593

107. Fernandez-Millan R, Medina-Merodio J-A, Plata R, Martinez-Herraiz J-J, Gutierrez-Martinez J-M. A laboratory test expert system for clinical diagnosis support in primary health care. *Applied Sciences*. 2015;5(3):222-240. doi:10.3390/app5030222

108. Ferreira-Santos D, Rodrigues PP. Improving diagnosis in obstructive sleep apnea with clinical data: a bayesian network approach. In: *2017 IEEE 30th International Symposium on Computer-Based Medical Systems (CBMS)*. Thessaloniki: IEEE; 2017:612-617. doi:10.1109/CBMS.2017.19

109. Flores CD, Marcelo FJ, Rosecler BM, Ana R, Helder C. Method for building a medical training simulator with bayesian networks: SimDeCS. In: *2nd KES International Conference on*

Innovation in Medicine and Healthcare. Vol 207. San Sebastian, Spain: Studies in Health Technology and Informatics; 2014:102–114. doi:10.3233/978-1-61499-474-9-102

110. Fok SC, Ng EYK, Thimm GL. Developing case-based reasoning for discovery of breast cancer. *Journal of Mechanics in Medicine and Biology*. 2003;3(3 & 4):231-245.

111. Font CR, Vas J, Rebello-Aguirre AC, et al. Subjective and objective (SVM) analysis of Brain perfusion SPECT in migraine patients treated with acupuncture. Preliminary results of a pragmatic randomised controlled trial. In: *Neurosciences: Clinical Science*. Vol 37. Eur J Nucl Med Mol Imagining; 2010:S391.

112. Forbes D, Wongthongtham P, Singh J. Development of patient-practitioner assistive communications (PPAC) ontology for type 2 diabetes management. In: *Proceedings of The Workshop on New Trends of Computational Intelligence in Health Applications*. Syndey, NSW: CEUR-WS; 2012:43-54.

113. Fourie D, Booysen MJ. Real time identification of heart sounds using selectional regional correlation of the time frequency domain. In: *2015 IEEE Symposium Series on Computational Intelligence*. Cape Town, South Africa: IEEE; 2015:1326-1330. doi:10.1109/SSCI.2015.189

114. Freitas Junior MG, Nicolosi DEC. Computer system to aid in diagnosing acute coronary syndromes. *Global Heart*. 2014;9(1):e274. doi:10.1016/j.gheart.2014.03.2208

115. Frost DW, Vembu S, Wang J, Tu K, Morris Q, Abrams HB. Using the electronic medical record to identify patients at high risk for frequent emergency department visits and high system costs. *The American Journal of Medicine*. 2017;130(5):601.e17-601.e22. doi:10.1016/j.amjmed.2016.12.008

116. Gajjar K, Trevisan J, Owens G, et al. Fourier-transform infrared spectroscopy coupled with a classification machine for the analysis of blood plasma or serum: a novel diagnostic approach for ovarian cancer. *The Analyst*. 2013;138(14):3917. doi:10.1039/c3an36654e

117. Gamlyn L, Needham P, Sopher SM, Harris TJ. The development of a neural network-based ambulatory ECG monitor. *Neural Computing & Applications*. 1999;8(3):273-278. doi:10.1007/s005210050030

118. Gaonkar AP, Kulkarni R, Caytiles RD, Iyengar NChSN. Classification of lower back pain disorder using multiple machine learning techniques and identifying degree of importance of each parameter. *International Journal of Advanced Science and Technology*. 2017;105:11-24. doi:10.14257/ijast.2017.105.02

119. García-Altés A, Santín D, Barenys M. Applying artificial neural networks to the diagnosis of organic dyspepsia. *Statistical Methods in Medical Research*. 2007;16(4):331-346. doi:10.1177/0962280206071839

120. Gharehbaghi A, Dutoit T, Sepehri AA, Kocharian A, Lindén M. A novel method for screening children with isolated bicuspid aortic valve. *Cardiovascular Engineering and Technology*. 2015;6(4):546-556. doi:10.1007/s13239-015-0238-6

121. Gharehbaghi A, Linden M, Babic A. A decision support system for cardiac disease diagnosis based on machine learning methods. *Studies in Health Technology and Informatics*. 2017:43–47. doi:10.3233/978-1-61499-753-5-43

122. Gharehbaghi A, Sepehri AA, Kocharian A, Lindén M. An intelligent method for discrimination between aortic and pulmonary stenosis using phonocardiogram. In: Jaffray DA, ed. *World Congress on Medical Physics and Biomedical Engineering, June 7-12, 2015, Toronto, Canada*. Vol 51. Cham: Springer International Publishing; 2015:1010-1013. doi:10.1007/978-3-319-19387-8_246

123. Giabbanelli PJ, Torsney-Weir T, Mago VK. A fuzzy cognitive map of the psychosocial determinants of obesity. *Applied Soft Computing*. 2012;12(12):3711-3724. doi:10.1016/j.asoc.2012.02.006

124. Gibbons RD, Hooker G, Finkelman MD, et al. The computerized adaptive diagnostic test for major depressive disorder (CAD-MDD): a screening tool for depression. *The Journal of Clinical Psychiatry*. 2013;74(07):669-674. doi:10.4088/JCP.12m08338

125. Glasspool DW, Fox J, Coulson AS, Emery J. Risk assessment in genetics: a semi-quantitative approach. *Stud Health Technol Inform*. 2001;2001(84):459-463.

126. Goh WL, Lau KT. ESMP - expert system for medicine prescription. *Expert Systems With Applications*. 1991;3:457-462.

127. Goldstein MK, Hoffman BB, Coleman RW, et al. Implementing clinical practice guidelines while taking account of changing evidence: ATHENA DSS, an easily modifiable decision-support system for managing hypertension in primary care. *AMIA*. 2000:300-304.

128. Goldstein MK, Coleman RW, Tu SW, et al. Translating research into practice: organizational issues in implementing automated decision support for hypertension in three medical centers. *Journal of the American Medical Informatics Association*. 2004;11(5):368-376. doi:10.1197/jamia.M1534

129. Gonzalez C, Bernd B, Lopez DM. Adaptive intelligent systems for pHealth - an architectural approach. *pHealth*. 2012:170–175. doi:10.3233/978-1-61499-069-7-170

130. Greenberg JN, Toledano A, Adrian JS, Gutkowicz-Krusin D, Chen SC. Biopsy performance of a multi-spectral computer vision system for melanoma compared to physicians. In: *Journal of Investigative Dermatology*. Vol 132. Pigmentation & Melanoma; 2012:S131. doi:10.1038/jid.2012.90

131. Grigull L, Lechner W, Petri S, et al. Diagnostic support for selected neuromuscular diseases using answer-pattern recognition and data mining techniques: a proof of concept multicenter prospective trial. *BMC Medical Informatics and Decision Making*. 2016;16(1). doi:10.1186/s12911-016-0268-5

132. Gu Y, Kennelly J, Warren J, Nathani P, Boyce T. Automatic detection of skin and subcutaneous tissue infections from primary care electronic medical records. *Studies in Health Technology and Informatics*. 2015:74–80. doi:10.3233/978-1-61499-558-6-74

133. Guilbert TW, Arndt B, Temte J, et al. The theory and application of UW eHealth-PHINEX, a clinical electronic health record–public health information exchange. 2012;111(3):11.

134. Gums T, Carter B, Foster E. Medication adherence: algorithms for success (MED-ALS). In: *Pharmacotherapy: The Journal of Human Pharmacology and Drug Therapy*. Vol 37. Pharmacotherapy; 2017:e136.

135. Gunčar G, Kukar M, Notar M, et al. An application of machine learning to haematological diagnosis. *Scientific Reports*. 2018;8(1). doi:10.1038/s41598-017-18564-8

136. Hao S, Geng S, Fan L, Chen J, Zhang Q, Li L. Intelligent diagnosis of jaundice with dynamic uncertain causality graph model. *Journal of Zhejiang University-SCIENCE B*. 2017;18(5):393-401. doi:10.1631/jzus.B1600273

137. Harding SM. Prediction formulae for sleep-disordered breathing: *Current Opinion in Pulmonary Medicine*. 2001;7(6):381-385. doi:10.1097/00063198-200111000-00003

138. Hashemi J, Spina TV, Tepper M, et al. Computer vision tools for the non-invasive assessment of autism-related behavioral markers. 2012. http://arxiv.org/abs/1210.7014. Accessed July 3, 2019.

139. Hashemi J, Spina TV, Tepper M, et al. A computer vision approach for the assessment of autism-related behavioral markers. In: *2012 IEEE International Conference on Development and Learning and Epigenetic Robotics (ICDL)*. San Diego, CA, USA: IEEE; 2012:1-7. doi:10.1109/DevLrn.2012.6400865

140. Hashmi Z, Zrimec T, Hopkins A. A framework for automatic medical information processing of referral letters for query formulation. *The Journal on Information Technology in Healthcare*. 2009;7(6):327-344.

141. Hasman A, Pop P, Winkens RAG, Blom JL. To test or not to test, that is the question. *Clinica Chimica Acta*. 1993;222:49-56.

142. Hazlehurst BL, Lawrence JM, Donahoo WT, et al. Automating assessment of lifestyle counseling in electronic health records. *American Journal of Preventive Medicine*. 2014;46(5):457-464. doi:10.1016/j.amepre.2014.01.001

143. Hazlehurst B, Mullooly J, Naleway A, Crane B. Detecting possible vaccination reactions in clinical notes. *AMIA*. 2005:306-310.

144. He H, Wang J, Graco W, Hawkins S. Application of neural networks to detection of medical fraud. *Expert Systems with Applications*. 1997;13(4):329-336. doi:10.1016/S0957-4174(97)00045-6

145. Heckerling P, Canaris G, Flach S, Tape T, Wigton R, Gerber B. Predictors of urinary tract infection based on artificial neural networks and genetic algorithms. *International Journal of Medical Informatics*. 2007;76(4):289-296. doi:10.1016/j.ijmedinf.2006.01.005

146. Hejlesen OK, Olesen KG, Dessau R, Beltoft I, Trangeled M. Decision support for diagnosis of lyme disease. *Connecting Medical Informatics and Bio-Informatics*. 2005:205-210.

147. Hertroijs DFL, Elissen AMJ, Brouwers MCGJ, et al. A risk score including body mass index, glycated haemoglobin and triglycerides predicts future glycaemic control in people with type 2 diabetes. *Diabetes, Obesity and Metabolism*. 2018;20(3):681-688. doi:10.1111/dom.13148

148. Homa L, Rose J, Hovmand PS, et al. A participatory model of the paradox of primary care. *The Annals of Family Medicine*. 2015;13(5):456-465. doi:10.1370/afm.1841

149. Hommersom A, Verwer S, Lucas PJF. Discovering probabilistic structures of healthcare processes. In: Riaño D, Lenz R, Miksch S, Peleg M, Reichert M, ten Teije A, eds. *Process Support and Knowledge Representation in Health Care*. Vol 8268. Cham: Springer International Publishing; 2013:53-67. doi:10.1007/978-3-319-03916-9_5

150. Horowitz N, Moshkowitz M, Halpern Z, Leshno M. Applying data mining techniques in the development of a diagnostics questionnaire for GERD. *Digestive Diseases and Sciences*. 2007;52(8):1871-1878. doi:10.1007/s10620-006-9202-5

151. Hosomura N, Malmasi S, Timerman D, et al. Decline of insulin therapy and delays in insulin initiation in people with uncontrolled diabetes mellitus. *Diabetic Medicine*. 2017;34(11):1599-1602. doi:10.1111/dme.13454

152. Hosomura N, Malmasi S, Timerman D, et al. Studying insulin decline by patients with diabetes. In: *What's New in Diabetes Epidemiology?* American Diabetes Association; 2017:1.

153. Hrabak KM, Campbell JR, Tu SW, McClure R, Weida RT. Creating interoperable guidelines: requirements of vocabulary standards in immunization decision support. *MEDINFO*. 2007:930-934.

154. Hripcsak G, Soulakis ND, Li L, et al. Syndromic surveillance using ambulatory electronic health records. *Journal of the American Medical Informatics Association*. 2009;16(3):354-361. doi:10.1197/jamia.M2922

155. Hung J, Posey J, Freedman R, Thorton T. Electronic surveillance of disease states: a preliminary study in electronic detection of respiratory diseases in a primary care setting. *AMIA*. 1998:688-692.

156. Hunt DL, Haynes RB, Morgan D. Using old technology to implement modern computer-aided decision support for primary diabetes care. *AMIA*. 2001:274-278.

157. Huseth O, Rost TB. Developing an annotated corpus of patient histories from the primary care health record. In: *2007 IEEE International Conference on Bioinformatics and Biomedicine Workshops*. San Jose, CA, USA: IEEE; 2007:165-173. doi:10.1109/BIBMW.2007.4425415

158. Hylan TR, Von Korff M, Saunders K, et al. Automated prediction of risk for problem opioid use in a primary care setting. *The Journal of Pain*. 2015;16(4):380-387. doi:10.1016/j.jpain.2015.01.011

159. Inbarani HH, Kumar SS, Azar AT, Hassanien AE. Soft rough sets for heart valve disease diagnosis. In: Hassanien AE, Tolba MF, Taher Azar A, eds. *Advanced Machine Learning Technologies and Applications*. Vol 488. Cham: Springer International Publishing; 2014:347-356. doi:10.1007/978-3-319-13461-1_33

160. Jagannathan V, Mullett CJ, Arbogast JG, et al. Assessment of commercial NLP engines for medication information extraction from dictated clinical notes. *International Journal of Medical Informatics*. 2009;78(4):284-291. doi:10.1016/j.ijmedinf.2008.08.006

161. Jammeh E, Carroll C, Pearson S, et al. Using NHS primary care data to identify undiagnosed dementia. In: *ABN*. Vol 86. Journal of Neurology, Neurosurgery & Psychiatry; 2015:e4. doi:10.1136/jnnp-2015-312379.44

162. Janssen KJM, Siccama I, Vergouwe Y, et al. Development and validation of clinical prediction models: marginal differences between logistic regression, penalized maximum likelihood estimation, and genetic programming. *Journal of Clinical Epidemiology*. 2012;65(4):404-412. doi:10.1016/j.jclinepi.2011.08.011

163. Jarvik JG, Gold LS, Tan K, et al. Long-term outcomes of a large, prospective observational cohort of older adults with back pain. *The Spine Journal*. 2018;18(9):1540-1551. doi:10.1016/j.spinee.2018.01.018

164. Jatobá A, Bellas HC, Koster I, et al. Supporting decision-making in patient risk assessment using a hierarchical fuzzy model. *Cognition, Technology & Work*. 2018;20(3):477-488. doi:10.1007/s10111-018-0475-1

165. Javed F, Venkatachalam PA, Hani AFM. Knowledge based system with embedded intelligent heart sound analyser for diagnosing cardiovascular disorders. *Journal of Medical Engineering & Technology*. 2007;31(5):341-350. doi:10.1080/03091900600887876

166. Johnson P, Tu S, Jones N. Achieving reuse of computable guideline systems. *MEDINFO*. 2001:99-103.

167. Johnson PD, Tu S, Booth N, Sugden B, Purves IN. Using scenarios in chronic disease management guidelines for primary care. *AMIA*. 2000:389-393.

168. Jordan P, Shedden-Mora MC, Löwe B. Predicting suicidal ideation in primary care: An approach to identify easily assessable key variables. *General Hospital Psychiatry*. 2018;51:106-111. doi:10.1016/j.genhosppsych.2018.02.002

169. Joudaki H, Rashidian A, Minaei-Bidgoli B, et al. Improving fraud and abuse detection in general physician claims: a data mining study. *International Journal of Health Policy and Management*. 2015;5(3):165-172. doi:10.15171/ijhpm.2015.196

170. Kabadi S, Lee A, Kuhn M, Gray D. Predictive model of Parkinson's Disease in large electronic health records database. *Value in Health*. 2016;19(3):A59. doi:10.1016/j.jval.2016.03.179

171. Kalankesh L, Weatherall J, Ba-Dhfari T, Buchan I, Brass A. Taming EHR data: using semantic similarity to reduce dimensionality. *Studies in Health Technology and Informatics*. 2013:52–56. doi:10.3233/978-1-61499-289-9-52

172. Kalatzis FG, Exarchos TP, Giannakeas N, et al. Point-of-care monitoring and diagnostics for rheumatoid arthritis and multiple sclerosis. In: *Advanced Topics in Scattering Theory and Biomedical Engineering*. Patras, Greece: WORLD SCIENTIFIC; 2010:98-105. doi:10.1142/9789814322034_0010

173. Kalina J, Seidl L, Zvara K, Grunfeldova H, Slovak D, Zvarova J. System for selecting relevant information for decision support. *Data and Knowledge for Medical Decision Support*. 2013:83-87. doi:10.24105/ejbi.2013.09.1.1

174. Katigari MR, Ayatollahi H, Malek M, Haghighi M. Fuzzy expert system for diagnosing diabetic neuropathy. *World Journal of Diabetes*. 2017;8(2):80. doi:10.4239/wjd.v8.i2.80

175. Kay E, Agarwal A. DropConnected neural networks trained on time-frequency and inter-beat features for classifying heart sounds. *Physiological Measurement*. 2017;38(8):1645-1657. doi:10.1088/1361-6579/aa6a3d

176. Kent RD, Preney PD, Snowdon AW, et al. Design and implementation of a primary health care services navigational system architecture. In: Watada J, Phillips-Wren G, Jain LC, Howlett RJ, eds. *Intelligent Decision Technologies*. Vol 10. Berlin, Heidelberg: Springer Berlin Heidelberg; 2011:743-752. doi:10.1007/978-3-642-22194-1_73

177. Kerr GS, Richards JS, Nunziato CA, et al. Measuring physician adherence with gout quality indicators: a role for natural language processing: natural language processing and gout quality indicators. *Arthritis Care & Research*. 2015;67(2):273-279. doi:10.1002/acr.22406

178. Khalili SP, LaNoue M. Population health at home: building a data ware-house and applying cluster analysis to identify patterns in healthcare utilization and multimorbidity among primary care patients. In: *Abstracts from the 2017 Society of General Internal Medicine Annual Meeting*. JGIM; 2017:S786.

179. Klann JG, Anand V, Downs SM. Patient-tailored prioritization for a pediatric care decision support system through machine learning. *J Am Med Inform Assoc*. 2013;20:e267-e274. doi:10.1136/amiajnl-2013-001865

180. Klausner S, Entacher K, Kranzer S, Flamm M, Sonnichsen A, Fritsch G. ProPath - a guideline based software for the implementation into the medical environment. In: *IEEE Canada International Humanitarian Technology Conference*. IEEE; 2014:6.

181. Kocharian A, Sepehri A-A, Janani A, Malakan-Rad E. Efficiency, sensitivity and specificity of automated auscultation diagnosis device for detection and discrimination of cardiac murmurs in children. *Iran J Pediatr*. 2013;23(4):6.

182. Koeling R, Tate AR, Carroll JA. Automatically estimating the incidence of symptoms recorded in GP free text notes. In: *Proceedings of the First International Workshop on Managing Interoperability and Complexity in Health Systems - MIXHS '11*. Glasgow, Scotland, UK: ACM Press; 2011:43. doi:10.1145/2064747.2064757

183. Kok MR, Boon ME, Schreiner-Kok PG, Hermans J, Grobbee DE, Kok LP. Less medical intervention after sharp demarcation of grade 1-2 cervical intraepithelial neoplasia smears by neural network screening. *Cancer Cytopathology*. 2001;93(3):6.

184. Kop R, Hoogendoorn M, Teije A ten, et al. Predictive modeling of colorectal cancer using a dedicated pre-processing pipeline on routine electronic medical records. *Computers in Biology and Medicine*. 2016;76:30-38. doi:10.1016/j.compbiomed.2016.06.019

185. Koskela T-H, Ryynanen O-P, Soini EJ. Risk factors for persistent frequent use of the primary health care services among frequent attenders: A Bayesian approach. *Scandinavian Journal of Primary Health Care*. 2010;28(1):55-61. doi:10.3109/02813431003690596

186. Kruse C, Eiken P, Vestergaard P. Clinical fracture risk evaluated by hierarchical agglomerative clustering. *Osteoporosis International*. 2017;28(3):819-832. doi:10.1007/s00198-016-3828-8

187. Kunhimangalam R, Ovallath S, Joseph PK. Computer aided diagnostic problem solving: Identification of peripheral nerve disorders. *IRBM*. 2013;34(3):244-251. doi:10.1016/j.irbm.2013.04.003

188. Kuntagod N, Mukherjee C. Mobile decision support system for outreach health worker. In: *2011 IEEE 13th International Conference on E-Health Networking, Applications and Services*. Columbia, MO, USA: IEEE; 2011:56-59. doi:10.1109/HEALTH.2011.6026786

189. Kwiatkowska M, Atkins MS, Ayas NT, Ryan CF, Street H. Telemedicine system for early assessment of obstructive sleep apnea. In: Banff; 2005:147-155.

190. Lacagnina V, Leto-Barone MS, La Piana S, La Porta G, Pingitore G, Di Lorenzo G. Comparison between statistical and fuzzy approaches for improving diagnostic decision making in patients with chronic nasal symptoms. *Fuzzy Sets and Systems*. 2014;237:136-150. doi:10.1016/j.fss.2013.10.013

191. Lacson R, Harris K, Brawarsky P, et al. Evaluation of an automated information extraction tool for imaging data elements to populate a breast cancer screening registry. *Journal of Digital Imaging*. 2015;28(5):567-575. doi:10.1007/s10278-014-9762-4

192. LaFreniere D, Zulkernine F, Barber D, Martin K. Using machine learning to predict hypertension from a clinical dataset. In: *2016 IEEE Symposium Series on Computational Intelligence (SSCI)*. Athens, Greece: IEEE; 2016:1-7. doi:10.1109/SSCI.2016.7849886

193. Lähteenmäki J, Leppänen J, Orsama A-L, et al. Remote patient monitoring system with decision support. *BioMed*. 2010:5.

194. Lange LL, Haak SW, Lincoln MJ, et al. Use of Iliad to improve diagnostic performance of nurse practitioner students. *Journal of Nursing Education*. 1997;36(1):36-45.

195. Lappenschaar M, Hommersom A, Lucas PJF, Lagro J, Visscher S. Multilevel Bayesian networks for the analysis of hierarchical health care data. *Artificial Intelligence in Medicine*. 2013;57(3):171-183. doi:10.1016/j.artmed.2012.12.007

196. Lappenschaar M, Hommersom A, Lucas PJF, et al. Multilevel temporal Bayesian networks can model longitudinal change in multimorbidity. *Journal of Clinical Epidemiology*. 2013;66(12):1405-1416. doi:10.1016/j.jclinepi.2013.06.018

197. Lappenschaar M, Hommersom A, Visscher S, Lucas PJF. Modeling inter-practice variation of disease interactions using multilevel bayesian networks. In: *AIME'11: The 13th Conference on Artificial Intelligence in Medicine, ProBiomed'11*. Bled Slovenia; 2011:15.

198. Larkin A, Healy C, Warters M. Simulation-based medical education: strategies to improve T2D management? In: *Endocrine Healthcare Delivery & Education II*. San Diego: Endocrine Society; 2015. doi:10.1210/endo-meetings.2015.AHPAA.11

199. Lau K, Wilkinson J, Moorthy R. A web-based prediction score for head and neck cancer referrals. *Clinical Otolaryngology*. 2018;43(4):1043-1049. doi:10.1111/coa.13098

200. Lavesson N. Predicting the risk of future hospitalization. In: *2010 Workshops on Database and Expert Systems Applications*. Bilbao, TBD, Spain: IEEE; 2010:120-124. doi:10.1109/DEXA.2010.43

201. Lavrač N, Bohanec M, Pur A, et al. Resource modeling and analysis of regional public health care data by means of knowledge technologies. In: Miksch S, Hunter J, Keravnou ET, eds. *Artificial Intelligence in Medicine*. Vol 3581. Berlin, Heidelberg: Springer Berlin Heidelberg; 2005:414-418. doi:10.1007/11527770_55

202. Leidy NK, Malley KG, Steenrod A, et al. Can we find undiagnosed, high-risk patients with COPD in primary care? Using random forests to identify best variable sets for COPD case identification. In: *COPD: Screening and Diagnostic Tools*. Vol 189. San Diego, CA: Am J Respir Crit Care Med; 2014:A2961.

203. Leidy NK, Malley KG, Steenrod AW, et al. Insight into best variables for COPD case identification: a random forests analysis. *Chronic Obstructive Pulmonary Diseases: Journal of the COPD Foundation*. 2016;3(1):406-418. doi:10.15326/jcopdf.3.1.2015.0144

204. Li C-M, Du Y-C, Wu J-X, et al. Synchronizing chaotification with support vector machine and wolf pack search algorithm for estimation of peripheral vascular occlusion in diabetes mellitus. *Biomedical Signal Processing and Control*. 2014;9:45-55. doi:10.1016/j.bspc.2013.10.001

205. Lidbury BA, Richardson AM, Badrick T. Assessment of machine-learning techniques on large pathology data sets to address assay redundancy in routine liver function test profiles. *Diagnosis*. 2015;2(1):41-51. doi:10.1515/dx-2014-0063

206. Lijun Pan, Xiaoting Fu, Fangfang Cai, Yu Meng, Changjiang Zhang. A compact electronic medical record system for regional clinics and health centers in China: Design and its application. In: *2016 IEEE International Conference on Bioinformatics and Biomedicine (BIBM)*. Shenzhen, China: IEEE; 2016:1010-1015. doi:10.1109/BIBM.2016.7822660

207. Lin C, Lin C-M, Li S-T, Kuo S-C. Intelligent physician segmentation and management based on KDD approach. *Expert Systems with Applications*. 2008;34(3):1963-1973. doi:10.1016/j.eswa.2007.02.038

208. Lin H-S, Talwar HS, Tarca AL, et al. Autoantibody approach for serum-based detection of head and neck cancer. *Cancer Epidemiology Biomarkers & Prevention*. 2007;16(11):2396-2405. doi:10.1158/1055-9965.EPI-07-0318

209. Linden A, Yarnold PR. Using data mining techniques to characterize participation in observational studies: Data mining in observational studies. *Journal of Evaluation in Clinical Practice*. 2016;22(6):839-847. doi:10.1111/jep.12515

210. Linnarsson R. Decision support for drug prescription integrated with computer-based patient records in primary care. *Medical Informatics*. 1993;18(2):131-142. doi:10.3109/14639239309034475

211. Lismont J, Janssens A-S, Odnoletkova I, vanden Broucke S, Caron F, Vanthienen J. A guide for the application of analytics on healthcare processes: A dynamic view on patient

pathways. *Computers in Biology and Medicine*. 2016;77:125-134. doi:10.1016/j.compbiomed.2016.08.007

212. Liu F, Antieau LD, Yu H. Toward automated consumer question answering: Automatically separating consumer questions from professional questions in the healthcare domain. *Journal of Biomedical Informatics*. 2011;44(6):1032-1038. doi:10.1016/j.jbi.2011.08.008

213. Long J, Yuan MJ, Poonawala R. An observational study to evaluate the usability and intent to adopt an artificial intelligence–powered medication reconciliation tool. *interactive Journal of Medical Research*. 2016;5(2):e14. doi:10.2196/ijmr.5462

214. Luther SL, McCart JA, Berndt DJ, et al. Improving identification of fall-related injuries in ambulatory care using statistical text mining. *American Journal of Public Health*. 2015;105(6):1168-1173. doi:10.2105/AJPH.2014.302440

215. MacRae J, Darlow B, McBain L, et al. Accessing primary care big data: the development of a software algorithm to explore the rich content of consultation records. *BMJ Open*. 2015;5(8):e008160. doi:10.1136/bmjopen-2015-008160

216. Maglogiannis I, Loukis E, Zafiropoulos E, Stasis A. Support Vectors Machine-based identification of heart valve diseases using heart sounds. *Computer Methods and Programs in Biomedicine*. 2009;95(1):47-61. doi:10.1016/j.cmpb.2009.01.003

217. Mahesh V, Ramakrishnan S. Assessment and classification of normal and restrictive respiratory conditions through pulmonary function test and neural network. *Journal of Medical Engineering & Technology*. 2007;31(4):300-304. doi:10.1080/03091900701233962

218. Majnarić-Trtica L, Vitale B. Systems biology as a conceptual framework for research in family medicine; use in predicting response to influenza vaccination. *Primary Health Care Research & Development*. 2011;12(04):310-321. doi:10.1017/S1463423611000089

219. Maramba ID, Davey A, Elliott MN, et al. Web-based textual analysis of free-text patient experience comments from a survey in primary care. *JMIR Medical Informatics*. 2015;3(2):e20. doi:10.2196/medinform.3783

220. Markaki M, Germanakis I, Stylianou Y. Automatic classification of systolic heart murmurs. In: *2013 IEEE International Conference on Acoustics, Speech and Signal Processing*. Vancouver, BC, Canada: IEEE; 2013:1301-1305. doi:10.1109/ICASSP.2013.6637861

221. Marling C, Wiley M, Bunescu R, Shubrook J, Schwartz F. Emerging applications for intelligent diabetes management. *AI Magazine*. 2012;33(2):67-78.

222. Martin CM, Vogel C, Grady D, et al. Implementation of complex adaptive chronic care: the Patient Journey Record system (PaJR): Complex adaptive chronic care - PaJR. *Journal of Evaluation in Clinical Practice*. 2012;18(6):1226-1234. doi:10.1111/j.1365-2753.2012.01880.x

223. Martin L. Knowledge acquisition and evaluation of an expert system for managing disorders of the outer eye. *Computers in Nursing*. 2001;19(3):114-117.

224. Martinez FJ, Mannino DM, Leidy NK, et al. A new approach for identifying patients with undiagnosed, clinically significant COPD in primary care. In: *Not a Second Time: Solutions to COPD Readmissions and Care Management*. Vol 191. American Journal of Respiratory and Critical Care Medicine; 2015:A6311.

225. Martinez F, Mannino D, Leidy N, et al. Identifying patients with undiagnosed clinically-significant COPD in primary care: what questions should we be asking? In: *Obstructive Lung Diseases*. Vol 148. Chest; 2015:696A. doi:10.1378/chest.2273164

226. Martinez F, Mannino DM, Leidy NK, et al. A novel approach for identifying patients with undiagnosed clinically-significant COPD. In: *1.6 General Practice and Primary Care*. Vol 46. European Respiratory Society; 2015:OA3284. doi:10.1183/13993003.congress-2015.OA3284

227. Maslekar S, Gardiner AB, Monson JRT, Duthie GS. Artificial neural networks to predict presence of significant pathology in patients presenting to routine colorectal clinics: neural networks to predict colorectal pathology. *Colorectal Disease*. 2010;12(12):1254-1259. doi:10.1111/j.1463-1318.2009.02005.x

228. Masood A, Al-Jumaily A. SA-SVM based automated diagnostic system for skin cancer. In: Wang Y, Jiang X, Zhang D, eds. *Sixth International Conference on Graphic and Image Processing (ICGIP 2014)*. Beijing, China; 2015:94432L. doi:10.1117/12.2179094

229. Masood A, Al-Jumaily AA, Adnan T. Development of automated diagnostic system for skin cancer: performance analysis of neural network learning algorithms for classification. In: Wermter S, Weber C, Duch W, et al., eds. *Artificial Neural Networks and Machine Learning – ICANN 2014*. Vol 8681. Cham: Springer International Publishing; 2014:837-844. doi:10.1007/978-3-319-11179-7_105

230. Masood A, Al-Jumaily A, Anam K. Texture analysis based automated decision support system for classification of skin cancer using SA-SVM. In: Loo CK, Yap KS, Wong KW, Teoh A, Huang K, eds. *Neural Information Processing*. Vol 8835. Cham: Springer International Publishing; 2014:101-109. doi:10.1007/978-3-319-12640-1_13

231. Masood A, Al-Jumaily A, Aung YM. Scaled conjugate gradient based decision support system for automated diagnosis of skin cancer. In: *Biomedical Engineering / 817: Robotics Applications*. Zurich, Switzerland: ACTAPRESS; 2014. doi:10.2316/P.2014.818-020

232. Matheny ME, FitzHenry F, Speroff T, et al. Detection of infectious symptoms from VA emergency department and primary care clinical documentation. *International Journal of Medical Informatics*. 2012;81(3):143-156. doi:10.1016/j.ijmedinf.2011.11.005

233. Mathias JS, Agrawal A, Feinglass J, Cooper AJ, Baker DW, Choudhary A. Development of a 5 year life expectancy index in older adults using predictive mining of electronic health record

data. *Journal of the American Medical Informatics Association*. 2013;20(e1):e118-e124. doi:10.1136/amiajnl-2012-001360

234. Maurer A, Hanrahan S, Nedrud J, Hebb AO, Papandreou-Suppappola A. Suppression of neurostimulation artifacts and adaptive clustering of Parkinson's patients behavioral tasks using EEG. In: *2016 50th Asilomar Conference on Signals, Systems and Computers*. Pacific Grove, CA, USA: IEEE; 2016:851-855. doi:10.1109/ACSSC.2016.7869169

235. McBride J, Zhao X, Munro N, Jicha G, Smith C, Jiang Y. Discrimination of mild cognitive impairment and alzheimer's disease using transfer entropy measures of scalp EEG. *Journal of Healthcare Engineering*. 2015;6(1):55-70. doi:10.1260/2040-2295.6.1.55

236. McCart JA, Berndt DJ, Jarman J, Finch DK, Luther SL. Finding falls in ambulatory care clinical documents using statistical text mining. *Journal of the American Medical Informatics Association*. 2013;20(5):906-914. doi:10.1136/amiajnl-2012-001334

237. McFadden P, Crim A. Comparison of the effectiveness of interactive didactic lecture versus online simulation-based CME programs directed at improving the diagnostic capabilities of primary care practitioners. *Journal of Continuing Education in the Health Professions*. 2016;36(1):32-37. doi:10.1097/CEH.0000000000000061

238. Messadi M, Bessaid A, Taleb-Ahmed A. Extraction of specific parameters for skin tumour classification. *Journal of Medical Engineering & Technology*. 2009;33(4):288-295. doi:10.1080/03091900802451315

239. Meulendijk MC, Spruit MR, Brinkkemper S. Risk mediation in association rules. In: ten Teije A, Popow C, Holmes JH, Sacchi L, eds. *Artificial Intelligence in Medicine*. Vol 10259. Cham: Springer International Publishing; 2017:327-331. doi:10.1007/978-3-319-59758-4_38

240. Meulendijk M, Spruit M, Lefebvre A, Brinkkemper S. To what extent can prescriptions be meaningfully exchanged between primary care terminologies? A case study of four western European classification systems. *IET Software*. 2017;11(5):256-264. doi:10.1049/iet-sen.2016.0301

241. Meyer KE, Sather-Levine B, Laurent-Bopp D, Gruenewald D, Nichol P, Kimmerle M. The impact of clinical information systems research on the future of advanced practice nursing. *Adv Prac Nurs Q*. 1996;2(3):58-64.

242. Mezghani N, Chav R, Humbert L, Parent S, Skalli W, de Guise JA. A computer-based classifier of three-dimensional spinal scoliosis severity. *International Journal of Computer Assisted Radiology and Surgery*. 2008;3(1-2):55-60. doi:10.1007/s11548-008-0163-3

243. Michalowski M, Michalowski W, O'Sullivan D, Wilk S, Carrier M. AFGuide system to support personalized management of atrial fibrillation. *Association for the Advancement of Artificial Intelligence*. 2017.

244. Michelson M, Minton SN, See K. A language-modeling approach to health data interoperability. *Association for the Advancement of Artificial Intelligence*. 2014:8.

245. Mikołajczyk A, Kwasigroch A, Grochowski M. Intelligent system supporting diagnosis of malignant melanoma. In: Mitkowski W, Kacprzyk J, Oprzędkiewicz K, Skruch P, eds. *Trends in Advanced Intelligent Control, Optimization and Automation*. Vol 577. Cham: Springer International Publishing; 2017:828-837. doi:10.1007/978-3-319-60699-6_79

246. Miller RE, Learned-Miller EG, Trainer P, Paisley A, Blanz V. Early diagnosis of acromegaly: computers vs clinicians: Computer diagnosis of acromegaly. *Clinical Endocrinology*. 2011;75(2):226-231. doi:10.1111/j.1365-2265.2011.04020.x

247. Millier A, Mejri S, Aballea S A, Toumi M. Health care organizations/services clustering: comparison of several techniques using UK data. In: *Value in Health*. Vol 15. 2012:A532. doi:10.1016/j.jval.2012.08.1852

248. Mishra S, Yamasaki T, Imaizumi H. Supervised classification of dermatological diseases by deep learning. 2018. http://arxiv.org/abs/1802.03752. Accessed July 3, 2019.

249. Modell M, Iliffe S, Austin A, Leaning MS. From guidelines to decision support in the management of asthma. *Health Telematics for Clinical Guidelines and Protocols*. 1995:105-113.

250. Moraes E, Brito K, Meira S. CompoPHC: An ontology-based component for primary health care. In: *2012 IEEE 13th International Conference on Information Reuse & Integration (IRI)*. Las Vegas, NV, USA: IEEE; 2012:592-599. doi:10.1109/IRI.2012.6303063

251. Moran W, Zhang MJ, Su Z, Taber D, Mauldin P, Srinivas T. Using predictive modeling to manage cardiovascular risk and improve kidney transplant outcomes. In: *Cardiovascular Complications in Kidney Transplantation*. 2016:208.

252. Moretz C, Zhou Y, Dhamane AD, et al. Development and validation of a predictive model to identify individuals likely to have undiagnosed chronic obstructive pulmonary disease using an administrative claims database. *Journal of Managed Care & Specialty Pharmacy*. 2015;21(12):1149-1159. doi:10.18553/jmcp.2015.21.12.1149

253. Murtaugh MA, Gibson BS, Redd D, Zeng-Treitler Q. Regular expression-based learning to extract bodyweight values from clinical notes. *Journal of Biomedical Informatics*. 2015;54:186-190. doi:10.1016/j.jbi.2015.02.009

254. Myburgh HC, Jose S, Swanepoel DW, Laurent C. Towards low cost automated smartphone- and cloud-based otitis media diagnosis. *Biomedical Signal Processing and Control*. 2018;39:34-52. doi:10.1016/j.bspc.2017.07.015

255. Myburgh HC, van Zijl WH, Swanepoel D, Hellström S, Laurent C. Otitis media diagnosis for developing countries using tympanic membrane image-analysis. *EBioMedicine*. 2016;5:156-160. doi:10.1016/j.ebiom.2016.02.017

256. Nahar J, Ali ABMS, Imam T, Tickle K, Chen P. Brain cancer diagnosis-association rule based computational intelligence approach. In: *2016 IEEE International Conference on Computer and Information Technology (CIT)*. Nadi, Fiji: IEEE; 2016:89-95. doi:10.1109/CIT.2016.106

257. Newcomer SR, Steiner JF, Bayliss EA. Identifying subgroups of complex patients with cluster analysis. *The American Journal of Managed Care*. 2011;17(8):e324-e332.

258. Ng K, Stewart WF, Defilippi C, et al. Data driven modeling of electronic health record data to detect pre-diagnostic heart failure in primary care. In: *Health Tech*. Vol 132. Circulation; 2015:A17713.

259. Ng K, Steinbuhl S, deFilippi C, Dey S, Stewart W. Early detection of heart failure using electronic health records: practical implications for time before diagnosis, data diversity, data quantity, and data density. *Journal of Patient-Centered Research and Reviews*. 2017;4(3):174-175. doi:10.17294/2330-0698.1523

260. Ni L, Lu C, Liu N, Liu J. MANDY: towards a smart primary care chatbot application. In: Chen J, Theeramunkong T, Supnithi T, Tang X, eds. *Knowledge and Systems Sciences*. Vol 780. Singapore: Springer Singapore; 2017:38-52. doi:10.1007/978-981-10-6989-5_4

261. Nijeweme-d'Hollosy W, van Velsen L, Poel M, Groothuis-Oudshoorn CGM, Soer R, Hermens H. Evaluation of three machine learning models for self-referral decision support on low back pain in primary care. *International Journal of Medical Informatics*. 2018;110:31-41. doi:10.1016/j.ijmedinf.2017.11.010

262. Nijeweme-dHollosy WO, Velsen L van, Groothuis-Oudshoorn KGM, Soer R, Hermens H. Should I see a healthcare professional or can I perform self-care: self-referral decision support for patients with low back pain. In: *2016 IEEE International Conference on Healthcare Informatics (ICHI)*. Chicago, IL, USA: IEEE; 2016:495-503. doi:10.1109/ICHI.2016.90

263. Nikolova I, Boytcheva S, Angelova G, Angelov Z. Combining structured and free textual data of diabetic patients' smoking status. In: Dichev C, Agre G, eds. *Artificial Intelligence: Methodology, Systems, and Applications*. Vol 9883. Cham: Springer International Publishing; 2016:57-67. doi:10.1007/978-3-319-44748-3_6

264. O'Bryant SE, Edwards M, Johnson L, et al. A blood screening test for Alzheimer's disease. *Alzheimer's & Dementia: Diagnosis, Assessment & Disease Monitoring*. 2016;3:83-90. doi:10.1016/j.dadm.2016.06.004

265. O'Bryant SE, Xiao G, Zhang F, et al. Validation of a serum screen for Alzheimer's Disease across assay platforms, species, and tissues. *Journal of Alzheimer's Disease*. 2014;42(4):1325-1335. doi:10.3233/JAD-141041

266. Odunukan OW, Elayavilli RK, Cha S, et al. Natural language processing identifies important side-effects In very elderly patients With hypertension. In: Vol 64. Hypertension; 2014:A239.

267. Orient JM. Evaluation of abdominal pain: clinicians' performance compared with three protocols. *South Med J*. 1986;79(7):793-799.

268. Orsi T, Araujo E, Simoes R. Fuzzy chest pain assessment for unstable angina based on Braunwald symptomatic and obesity clinical conditions. In: *2014 IEEE International Conference on Fuzzy Systems (FUZZ-IEEE)*. Beijing, China: IEEE; 2014:1076-1082. doi:10.1109/FUZZ-IEEE.2014.6891836

269. Ou MH, West GAW, Lazarescu M, Clay CD. Evaluation of TELEDERM for dermatological services in rural and remote areas. *Artificial Intelligence in Medicine*. 2008;44(1):27-40. doi:10.1016/j.artmed.2008.04.006

270. Pakhomov SVS, Shah ND, Van Houten HK, Hanson PL, Smith SA. The role of the electronic medical record in the assessment of health related quality of life. *AMIA Annu Symp Proc*. 2011;2011:1080-1088.

271. Passi K, Zhao H. A decision support system (DSS) for colorectal cancer follow-up program via a semantic framework: *International Journal of Healthcare Information Systems and Informatics*. 2015;10(1):17-38. doi:10.4018/IJHISI.2015010102

272. Perlini S, Piepoli M, Marti G, et al. Treatment of chronic heart failure: an expert system advisor for general practitioners (*). *Acta Cardiologica*. 1990;XLV(5):365-378.

273. Perveen S, Shahbaz M, Guergachi A, Keshavjee K. Performance analysis of data mining classification techniques to predict diabetes. *Procedia Computer Science*. 2016;82:115-121. doi:10.1016/j.procs.2016.04.016

274. Pietilä A, El-Segaier M, Vigário R, Pesonen E. Blind source separation of cardiac murmurs from heart recordings. In: Rosca J, Erdogmus D, Príncipe JC, Haykin S, eds. *Independent Component Analysis and Blind Signal Separation*. Vol 3889. Berlin, Heidelberg: Springer Berlin Heidelberg; 2006:470-477. doi:10.1007/11679363_59

275. Piury J, Laita LM, Roanes-Lozano E, et al. A Gröbner bases-based rule based expert system for fibromyalgia diagnosis. *Revista de la Real Academia de Ciencias Exactas, Fisicas y Naturales Serie A Matematicas*. 2012;106(2):443-456. doi:10.1007/s13398-012-0064-8

276. Pollettini JT, Nicolas FP, Panico SG, et al. A software architecture-based framework supporting suggestion of medical surveillance level from classification of electronic patient records. In: *2009 International Conference on Computational Science and Engineering*. Vancouver, BC, Canada: IEEE; 2009:166-173. doi:10.1109/CSE.2009.231

277. Poulin C, Shiner B, Thompson P, et al. Predicting the risk of suicide by analyzing the text of clinical notes. Brusic V, ed. *PLoS ONE*. 2014;9(1):e85733. doi:10.1371/journal.pone.0085733

278. Prasanna P, Jain S, Bhagat N, Madabhushi A. Decision support system for detection of diabetic retinopathy using smartphones. In: *Proceedings of the ICTs for Improving Patients Rehabilitation Research Techniques*. Venice, Italy: IEEE; 2013. doi:10.4108/icst.pervasivehealth.2013.252093

279. Ptochos D, Panopoulos D, Metaxiotis K, Askounis D, Psarras J. Using internet GIS technology for early warning, response and controlling the quality of the public health sector. *International Journal of Electronic Healthcare*. 2004;1(1):78. doi:10.1504/IJEH.2004.004661

280. Quiceno-Manrique AF, Godino-Llorente JI, Blanco-Velasco M, Castellanos-Dominguez G. Selection of dynamic features based on time–frequency representations for heart murmur detection from phonocardiographic signals. *Annals of Biomedical Engineering*. 2010;38(1):118-137. doi:10.1007/s10439-009-9838-3

281. Rahimi A, Liaw S-T, Taggart J, Ray P, Yu H. Validating an ontology-based algorithm to identify patients with Type 2 Diabetes Mellitus in electronic health records. *International Journal of Medical Informatics*. 2014;83(10):768-778. doi:10.1016/j.ijmedinf.2014.06.002

282. Rajan V, Bhattacharya S, Shetty R, Sitaram A, Vivek G. Clinical decision support for stroke using multi–view learning based models for NIHSS scores. In: Cao H, Li J, Wang R, eds. *Trends and Applications in Knowledge Discovery and Data Mining*. Vol 9794. Cham: Springer International Publishing; 2016:190-199. doi:10.1007/978-3-319-42996-0_16

283. Rajkomar A, Yim JWL, Grumbach K, Parekh A. Weighting primary care patient panel size: a novel electronic health record-derived measure using machine learning. *JMIR Medical Informatics*. 2016;4(4):e29. doi:10.2196/medinform.6530

284. Raluy-Callado M, Cox A, MacLachlan S, Gabriel S, Dinet J. Resource utilisation and costs in patients with post-stroke spasticity In the United Kingdom. *Value in Health*. 2015;18(7):A386. doi:10.1016/j.jval.2015.09.840

285. Raluy-Callado M, Cox A, MacLachlan S, et al. A retrospective study to assess resource utilization and costs in patients with post-stroke spasticity in the United Kingdom. *Current Medical Research and Opinion*. 2018;34(7):1317-1324. doi:10.1080/03007995.2018.1447449

286. Rao RR, Makkithaya K, Gupta N. Ontology based semantic representation for Public Health data integration. In: *2014 International Conference on Contemporary Computing and Informatics (IC3I)*. Mysore, India: IEEE; 2014:357-362. doi:10.1109/IC3I.2014.7019701

287. Ray HN, Boxwala AA, Anantraman V, Ohno-Machado L. Providing context-sensitive decision-support based on WHO guidelines. *AMIA*. 2002:637-641.

288. Reece AG, Danforth CM. Instagram photos reveal predictive markers of depression. *EPJ Data Science*. 2017;6(1). doi:10.1140/epjds/s13688-017-0110-z

289. Reed C, Boswell B, Neville R. Multi-agent patient representation in primary care. In: Miksch S, Hunter J, Keravnou ET, eds. *Artificial Intelligence in Medicine*. Vol 3581. Berlin, Heidelberg: Springer Berlin Heidelberg; 2005:375-384. doi:10.1007/11527770_50

290. Rezaeiahari M, Poranki S, Khasawneh M. Predicting emergency department visits among patients receiving primary care using risk scores. In: *Proceedings of the 2017 Industrial and Systems Engineering Conference*. ; 2017:6.

291. Riaño D, Fernández-Pérez A. Simulation-based episodes of care data synthetization for chronic disease patients. In: Riaño D, Lenz R, Reichert M, eds. *Knowledge Representation for Health Care*. Vol 10096. Cham: Springer International Publishing; 2017:36-50. doi:10.1007/978-3-319-55014-5_3

292. Rodbard D, Vigersky RA. Design and development of a computer assisted clinical decision support system to help physicians manage patients with type 2 diabetes mellitus. In: *Tools for Improving Diabetes Control*. Vol 53. Diabetologia; 2010:S414. doi:10.1007/s00125-010-1872-z

293. Rodríguez-Solano C, Laita LM, Roanes-Lozano E, López-Corral L, Laita L. A computational system for diagnosis of depressive situations. *Expert Systems with Applications*. 2006;31(1):47-55. doi:10.1016/j.eswa.2005.09.011

294. Romero-Córdoba R, Olivas JA, Romero FP, Alonso-Gonzalez F, Serrano-Guerrero J. An application of fuzzy prototypes to the diagnosis and treatment of fuzzy diseases: diagnosis and treatment of fuzzy diseases. *International Journal of Intelligent Systems*. 2017;32(2):194-210. doi:10.1002/int.21836

295. Røst TB, Sørby ID, Seland G. Development of a medication reconciliation tool for Norwegian primary care EPR systems: experiences from a user-initiated project. In: *Proceedings of the 2nd European Workshop on Practical Aspects of Health Informatics*. Trondheim, Norway: ceur-ws; 2014:10.

296. Rother A-K, Schwerk N, Brinkmann F, Klawonn F, Lechner W, Grigull L. Diagnostic support for selected paediatric pulmonary diseases using answer-pattern recognition in questionnaires based on combined data mining applications—a monocentric observational pilot study. Hartl D, ed. *PLOS ONE*. 2015;10(8):e0135180. doi:10.1371/journal.pone.0135180

297. Saadé RG, Tsoukas A, Tsoukas G. Prototyping a decision support system in the clinical environment: assessment of patients with osteoporosis OSTEODSS. *Expert Systems with Applications*. 2004;27(3):427-438. doi:10.1016/j.eswa.2004.05.019

298. Safarova MS, Liu H, Kullo IJ. Rapid identification of familial hypercholesterolemia from electronic health records: The SEARCH study. *Journal of Clinical Lipidology*. 2016;10(5):1230-1239. doi:10.1016/j.jacl.2016.08.001

299. Safran C, Rind DM, Davis RB, et al. A clinical trial of a knowledge-based medical record. *MEDINFO*. 1995;95:1076-1080.

300. Safran C, Rind DM, Davis RM, et al. An electronic medical record that helps care for patients with HIV infection. *AMIA*. 1994:224-228.

301. Salama MA, Soliman OS, Maglogiannis I, Hassanien AE, Fahmy AA. Rough set-based identification of heart valve diseases using heart sounds. In: Skowron A, Suraj Z, eds. *Rough Sets and Intelligent Systems - Professor Zdzisław Pawlak in Memoriam*. Vol 43. Berlin, Heidelberg: Springer Berlin Heidelberg; 2013:475-491. doi:10.1007/978-3-642-30341-8_25

302. Sali R, Roohafza H, Sadeghi M, Andalib E, Shavandi H, Sarrafzadegan N. Validation of the revised stressful life event questionnaire using a hybrid model of genetic algorithm and artificial neural networks. *Computational and Mathematical Methods in Medicine*. 2013;2013:1-7. doi:10.1155/2013/601640

303. Salmeron JL, Rahimi SA, Navali AM, Sadeghpour A. Medical diagnosis of Rheumatoid Arthritis using data driven PSO–FCM with scarce datasets. *Neurocomputing*. 2017;232:104-112. doi:10.1016/j.neucom.2016.09.113

304. Saraiva R, Perkusich M, Silva L, Almeida H, Siebra C, Perkusich A. Early diagnosis of gastrointestinal cancer by using case-based and rule-based reasoning. *Expert Systems with Applications*. 2016;61:192-202. doi:10.1016/j.eswa.2016.05.026

305. Scala PL, Pasquale D, Tresoldi D, Lafortuna CL, Rizzo G, Padula M. Ontology-supported clinical profiling for the evaluation of obesity and related comorbidities. *Studies in Health Technology and Informatics*. 2012:1025–1029. doi:10.3233/978-1-61499-101-4-1025

306. Schilithz AOC, Kale PL, Gama SGN, Nobre FF. Risk groups in children under six months of age using self-organizing maps. *Computer Methods and Programs in Biomedicine*. 2014;115(1):1-10. doi:10.1016/j.cmpb.2014.02.011

307. Schilling C, Mortimer D, Dalziel K, Heeley E, Chalmers J, Clarke P. Using classification and regression trees (CART) to identify prescribing thresholds for cardiovascular disease. *PharmacoEconomics*. 2016;34(2):195-205. doi:10.1007/s40273-015-0342-3

308. Sen A, Sinha AP. An ontological model of the practice transformation process. *Journal of Biomedical Informatics*. 2016;61:298-318. doi:10.1016/j.jbi.2016.05.001

309. Seroussi B, Bouaud J, Dreau H, et al. ASTI: a guideline-based drug-ordering system for primary care. *MEDINFO*. 2001;2001:528-532.

310. Shalev V, Kinar Y, Kalkstein N, et al. Computational analysis of blood counts significantly increases detection rate of gastric and colorectal cancers. In: *Esophageal, Gastric and Duodenal Disorders*. Vol 28. Journal of Gastroenterology and Hepatology; 2013:761.

311. Shankar RD, Martins SB, Tu SW, Goldstein MK, Musen MA. Building an explanation function for a hypertension decision-support system. *MEDINFO*. 2001;2001:538-542.

312. Shavandi H, Mahlooji H. Fuzzy hierarchical queueing models for the location set covering problem in congested systems. *Scientia Iranica*. 2008;15(3):378-388.

313. Shen Y, Yuan K, Chen D, et al. An ontology-driven clinical decision support system (IDDAP) for infectious disease diagnosis and antibiotic prescription. *Artificial Intelligence in Medicine*. 2018;86:20-32. doi:10.1016/j.artmed.2018.01.003

314. Siepmann JP, Bachman JW. HTN-APT: computer aid in hypertension management. *The Journal of Family Practice*. 1987;24(3):313-316.

315. Simon A. A multivariate portable noninvasive diagnostic approach to actively assess cognitive function in Alzheimer's disease (AD) and mild cognitive impairment (MCI). *Alzheimer's & Dementia*. 2012;8(4):115-116. doi:10.1016/j.jalz.2012.05.299

316. Simon A. A multi-modal biosensor-based approach to aid in the diagnosis of concussion and Alzheimer's Disease. In: Vol 10. Alzheimer's & Dementia; 2014:662. doi:10.1016/j.jalz.2014.05.1181

317. Simon A, Ashrafiuon H, Ghorbanian P, Devilbiss D. A noninvasive device-based approach to aid in the diagnosis of mTBI and Alzheimer's disease: Preliminary findings from Clinical Pilot Studies. *Alzheimer's & Dementia*. 2013;9(4):P222. doi:10.1016/j.jalz.2013.05.417

318. Simon A, Devilbiss D. Multivariate models of biosensor data to actively assess sports concussion and mild traumatic brain injury. In: Vol 86. Neurology; 2016.

319. Sinnott MM, Carr B, Markey J, et al. Knowledge based lipid management system for general practitioners. *Clinica Chimica Acta*. 1993;222(1-2):71-77. doi:10.1016/0009-8981(93)90093-J

320. Sola D, Borioli GS, Quaglia R. Predicting GPs' engagement with artificial intelligence. *British Journal of Healthcare Management*. 2018;24(3):134-140. doi:10.12968/bjhc.2018.24.3.134

321. Soler JK, Corrigan D, Kazienko P, et al. Evidence-based rules from family practice to inform family practice; the learning healthcare system case study on urinary tract infections. *BMC Family Practice*. 2015;16(63). doi:10.1186/s12875-015-0271-4

322. Solms F, Smit E, Nel ZJ. A neural network diagnostic tool for the chronic fatigue syndrome. In: *Proceedings of International Conference on Neural Networks (ICNN'96)*. Vol 2. Washington, DC, USA: IEEE; 1996:778-781. doi:10.1109/ICNN.1996.548995

323. Song MH, Lee YH, Kang UG. Comparison of machine learning algorithms for classification of the sentences in three clinical practice guidelines. *Healthcare Informatics Research*. 2013;19(1):16. doi:10.4258/hir.2013.19.1.16

324. Sourla E, Syrimpeis V, Stamatopoulou K-M, Merekoulias G, Tsakalidis A, Tzimas G. Exploiting fuzzy expert systems in cardiology. In: Iliadis L, Papadopoulos H, Jayne C, eds. *Engineering Applications of Neural Networks*. Vol 384. Berlin, Heidelberg: Springer Berlin Heidelberg; 2013:80-89. doi:10.1007/978-3-642-41016-1_9

325. South BR, Shen S, Jones M, et al. Developing a manually annotated clinical document corpus to identify phenotypic information for inflammatory bowel disease. *BMC Bioinformatics*. 2009;10(S9). doi:10.1186/1471-2105-10-S9-S12

326. Spyropoulos J, Boutsalis G, Chatterjee P, LaCouture M. Improving clinical decisions in dyslipidemia: simulation-based medical education in action. In: *Innovations in Practice Management and Social Media*. Vol 69. Journal of the American College of Cardiology; 2017:2502. doi:10.1016/S0735-1097(17)35891-6

327. Srinivas S, Ravindran AR. Optimizing outpatient appointment system using machine learning algorithms and scheduling rules: A prescriptive analytics framework. *Expert Systems with Applications*. 2018;102:245-261. doi:10.1016/j.eswa.2018.02.022

328. St-Maurice J, Kuo M-H, Gooch P. A proof of concept for assessing emergency room use with primary care data and natural language processing. *Methods of Information in Medicine*. 2013;52(01):33-42. doi:10.3414/ME12-01-0012

329. St-Maurice J, Kuo MH. Analyzing primary care data to characterize inappropriate emergency room use. *Studies in Health Technology and Informatics*. 2012:990–994. doi:10.3233/978-1-61499-101-4-990

330. Stan O, Avram C, Stefan I, Astilean A. Integrated innovative solutions to improve healthcare scheduling. In: *2016 IEEE International Conference on Automation, Quality and Testing, Robotics (AQTR)*. Cluj-Napoca, Romania: IEEE; 2016:1-6. doi:10.1109/AQTR.2016.7501301

331. Stanganelli I, Brucale A, Calori L, et al. Computer-aided diagnosis of melanocytic lesions. *Anticancer Res*. 2005;25:4577-4582.

332. Šter M, Švab I, Šter B. Prediction of intended career choice in family medicine using artificial neural networks. *European Journal of General Practice*. 2015;21(1):63-69. doi:10.3109/13814788.2014.933314

333. Stiglic G, Kokol P. Intelligent patient and nurse scheduling in ambulatory health care centers. In: *2005 IEEE Engineering in Medicine and Biology 27th Annual Conference*. Shanghai, China: IEEE; 2005:5475-5478. doi:10.1109/IEMBS.2005.1615722

334. Suhasini A, Palanivel S, Ramalingam V. Multimodel decision support system for psychiatry problem. *Expert Systems with Applications*. 2011;38(5):4990-4997. doi:10.1016/j.eswa.2010.09.152

335. Sumner 2nd W, Hagen MD, Rovinelli R. The item generation methodology of an empiric simulation project. *Adv Health Sci Educ*. 1999;4:49-66.

336. Sumner 2nd W, Truszczynski M, Marek VM. Simulating patients with parallel health state networks. *AMIA*. 1998:438-442.

337. Sumner 2nd. W, Xu JZ. Modeling fatigue. In: *AMIA Annual Symposium Proceedings*. 2002:747-751.

338. Sumner 2nd. W, Xu JZ, Roussel G, Hagen MD. Modeling relief. In: *AMIA Annual Symposium Proceedings*. 2007:706-710.

339. Syed Z, Leeds D, Curtis D, Nesta F, Levine RA, Guttag J. A framework for the analysis of acoustical cardiac signals. *IEEE Transactions on Biomedical Engineering*. 2007;54(4):651-662. doi:10.1109/TBME.2006.889189

340. Syiam MM. A neural network expert system for diagnosing eye diseases. In: *Proceedings of the Tenth Conference on Artificial Intelligence for Applications*. San Antonia, TX, USA: IEEE; 1994:491-492. doi:10.1109/CAIA.1994.323624

341. Szecsenyi J, Buschhorn A, Kochen M. General practitioners' attitudes towards future developments in practice computing—a representative survey in the north of Germany. *Family Practice*. 1992;9(3):357-361. doi:10.1093/fampra/9.3.357

342. Tan A, Liang MW, Chio MT, et al. Designing a mobile imaging system for early melanoma detection. In: *6th Singapore Health & Biomedical Congress*. Vol 44. Singapore: Annals of the Academy of Medicine; 2015:S187. doi:10.1016/j.jaad.2015.02.366

343. Tape TG, Rn RAS, Campbell JR. Implementing guidelines in ambulatory practice. *AMIA*. 1993:806-807.

344. Teipel SJ, Kilimann I, Dyrba M, Brüggen K, Thyrian JR, Hoffmann W. Structural connectivity as a biomarker for Alzheimer's disease: Evaluation in a multicenter trial and a primary care cohort. *Alzheimer's & Dementia*. 2015;11(7):P696. doi:10.1016/j.jalz.2015.06.1545

345. Tekin C, Atan O, Van Der Schaar M. Discover the expert: context-adaptive expert selection for medical diagnosis. *IEEE Transactions on Emerging Topics in Computing*. 2015;3(2):220-234. doi:10.1109/TETC.2014.2386133

346. Teles G, Oliveira C, Braga R, et al. Using Bayesian networks to improve the decision-making process in public health systems. In: *2014 IEEE 16th International Conference on E-*

Health Networking, Applications and Services (Healthcom). Natal: IEEE; 2014:565-570. doi:10.1109/HealthCom.2014.7001904

347. Thurin N, Castilloux AM, Reich C, Hermann R, Frise S, Moride Y. Assessing the feasibility and performance of the HAWK electronic medical records for drug safety surveillance. In: *Drug Safety*. Vol 38. Prague, Czech Republic: Drug Safety; 2015:1033. doi:10.1007/s40264-015-0346-0

348. Timpka T. Knowledge-based decision support for general practitioners: an integrated design. *Computer Methods and Programs in Biomedicine*. 1987;25:49-60.

349. Ting SL, Kwok SK, Tsang AHC, Lee WB. A hybrid knowledge-based approach to supporting the medical prescription for general practitioners: Real case in a Hong Kong medical center. *Knowledge-Based Systems*. 2011;24(3):444-456. doi:10.1016/j.knosys.2010.12.011

350. Tirunagari S, Bull SC, Vehtari A, Farmer C, de Lusignan S, Poh N. Automatic detection of acute kidney injury episodes from primary care data. In: *2016 IEEE Symposium Series on Computational Intelligence (SSCI)*. Athens, Greece: IEEE; 2016:1-6. doi:10.1109/SSCI.2016.7849885

351. Trevisan J, Angelov PP, Carmichael PL, Scott AD, Martin FL. Extracting biological information with computational analysis of Fourier-transform infrared (FTIR) biospectroscopy datasets: current practices to future perspectives. *The Analyst*. 2012;137(14):3202. doi:10.1039/c2an16300d

352. Trtica-Majnaric L, Zekic-Susac M, Sarlija N, Vitale B. Prediction of influenza vaccination outcome by neural networks and logistic regression. *Journal of Biomedical Informatics*. 2010;43(5):774-781. doi:10.1016/j.jbi.2010.04.011

353. Van der Niepen P, Woestenburg A, Brié H, et al. Effectiveness of valsartan for treatment of hypertension: patient profiling and hierarchical modeling of determinants and outcomes (the Preview Study). *Annals of Pharmacotherapy*. 2009;43(5):849-861. doi:10.1345/aph.1L576

354. Van Voorhees BW, Paunesku D, Gollan J, Kuwabara S, Reinecke M, Basu A. Predicting future risk of depressive episode in adolescents: The Chicago Adolescent Depression Risk Assessment (CADRA). *The Annals of Family Medicine*. 2008;6(6):503-511. doi:10.1370/afm.887

355. VanQuekelberghe P, Jakob T, Hoffmann D, Wetter T, Finkeissen E. Minimalist knowledge representation of primary care diseases in the medrapid.info knowledge base. *Journal of Innovation in Health Informatics*. 2005;13(4):239-248. doi:10.14236/jhi.v13i4.603

356. Vega R, Sanchez-Ante G, Falcon-Morales LE, Sossa H, Guevara E. Retinal vessel extraction using Lattice Neural Networks with dendritic processing. *Computers in Biology and Medicine*. 2015;58:20-30. doi:10.1016/j.compbiomed.2014.12.016

357. Velickovski F, Ceccaroni L, Marti R, et al. Automated spirometry quality assurance: supervised learning from multiple experts. *IEEE Journal of Biomedical and Health Informatics*. 2018;22(1):276-284. doi:10.1109/JBHI.2017.2713988

358. Vijayakrishnan R, Steinhubl SR, Ng K, et al. Prevalence of heart failure signs and symptoms in a large primary care population identified through the use of text and data mining of the electronic health record. *Journal of Cardiac Failure*. 2014;20(7):459-464. doi:10.1016/j.cardfail.2014.03.008

359. Viveros MS, Nearhos JP, Rothman MJ. Applying data mining techniques to a health insurance information system. In: *Proceedings of the 22nd VLDB Conference*. Mumbai (Bombay), India: VLDB; 1996:286-294.

360. Vos R, Aarts S, van Mulligen E, et al. Finding potentially new multimorbidity patterns of psychiatric and somatic diseases: exploring the use of literature-based discovery in primary care research. *JAMIA*. 2014;21(1):139-145. doi:10.1136/amiajnl-2012-001448

361. Walton R, Ilic Z. Knowledge engineering for drug prescribing guidelines. *Health Telematics for Clinical Guidelines and Protocols*. 1995:75-85.

362. Wang W, Richards G, Rea S. Hybrid data mining ensemble for predicting osteoporosis risk. In: *2005 IEEE Engineering in Medicine and Biology 27th Annual Conference*. Shanghai, China: IEEE; 2005:886-889. doi:10.1109/IEMBS.2005.1616557

363. Wang W, Rea S. Intelligent ensemble system aids osteoporosis early detection. In: *Proceedings of the 6th WSEAS Int. Conf. on Evolutionary Computing*. Lisbon, Portugal; 2005:123-128.

364. Wang X, Valdez TA, Bi J. Detecting tympanostomy tubes from otoscopic images via offline and online training. *Computers in Biology and Medicine*. 2015;61:107-118. doi:10.1016/j.compbiomed.2015.03.025

365. Wang Y, Patrick J, Miller G, O'Hallaran J. A computational linguistics motivated mapping of ICPC-2 PLUS to SNOMED CT. *BMC Medical Informatics and Decision Making*. 2008;8(S1). doi:10.1186/1472-6947-8-S1-S5

366. Wang Y, Chen H, Li R, Duan N, Lewis-Fernández R. Prediction-based structured variable selection through the receiver operating characteristic curves. *Biometrics*. 2011;67(3):896-905. doi:10.1111/j.1541-0420.2010.01533.x

367. Wang Z, Shah AD, Tate AR, Denaxas S, Shawe-Taylor J, Hemingway H. Extracting diagnoses and investigation results from unstructured text in electronic health records by semi-supervised machine learning. Brusic V, ed. *PLoS ONE*. 2012;7(1):e30412. doi:10.1371/journal.pone.0030412

368. Warren, Warren, Freedman. Interviewing expertise in primary care medicine: a knowledge-based support system. In: *Proceedings of the Twenty-Seventh Hawaii International Conference on System Sciences HICSS-94*. Wailea, HI, USA: IEEE Comput. Soc. Press; 1994:173-182. doi:10.1109/HICSS.1994.323354

369. Warren J, Beliakov G, van der Zwaag B. Fuzzy logic in clinical practice decision support systems. In: *Proceedings of the 33rd Annual Hawaii International Conference on System Sciences*. Vol vol.1. Maui, HI, USA: IEEE Comput. Soc; 2000:10. doi:10.1109/HICSS.2000.926789

370. Warren JR, Davidovic A, Spenceley S, Bolton P. Mediface: anticipative data entry interface for general practitioners. In: *Proceedings 1998 Australasian Computer Human Interaction Conference. OzCHI'98 (Cat. No.98EX234)*. Adelaide, SA, Australia: IEEE Comput. Soc; 1998:192-199. doi:10.1109/OZCHI.1998.732214

371. Warren JR, Posey B, Thornton T, ParangA P. Can computer autoacquisition of medical information meet the needs of the future? A feasibility study in direct computation of the fine grained electronic medical record. *AMIA*. 1999:445-449.

372. Warren JR, Tyerman SP. Webifying a patient interview support application. *Medical Informatics*. 1998;23(1):63-74. doi:10.3109/14639239809001392

373. Watrous RL, Thompson WR, Ackerman SJ. The impact of computer-assisted auscultation on physician referrals of asymptomatic patients with heart murmurs. *Clinical Cardiology*. 2008;31(2):79-83. doi:10.1002/clc.20185

374. Weng SF, Reps J, Kai J, Garibaldi JM, Qureshi N. Can machine-learning improve cardiovascular risk prediction using routine clinical data? Liu B, ed. *PLOS ONE*. 2017;12(4):e0174944. doi:10.1371/journal.pone.0174944

375. Wenner AR, Ferrante M, Belser D. Instant medical history. *AMIA*. 1994:1036.

376. Wickramasinghe K, Alahakoon D, Schattner P, Georgeff M. Self-organizing maps for translating health care knowledge: a case study in diabetes management. In: Wang D, Reynolds M, eds. *AI 2011: Advances in Artificial Intelligence*. Vol 7106. Berlin, Heidelberg: Springer Berlin Heidelberg; 2011:162-171. doi:10.1007/978-3-642-25832-9_17

377. Widmer G, Horn W, Nagele B. Automatic knowledge base refinement: Learning from examples and deep knowledge in rheumatology. *Artif Intell Med*. 1993;5(3):225-243. doi:10.1016/0933-3657(93)90026-Y

378. Witteman CL, Renooij S, Koele P. Medicine in words and numbers: a cross-sectional survey comparing probability assessment scales. *BMC Medical Informatics and Decision Making*. 2007;7(1). doi:10.1186/1472-6947-7-13

379. Wright A, Ricciardi TN, Zwick M. Application of information-theoretic data mining techniques in a national ambulatory practice outcomes research network. *AMIA*. 2005:829-833.

380. Wu J, Roy J, Stewart WF. Prediction modeling using EHR data. *Medical Care*. 2010;48(6):8.

381. Yamauchi K, Fukatsu T. A decision support system for diagnostic consultation in laboratory tests. *MEDINFO*. 1995;95:1034.

382. Yin Z, Zhao Y, Lu X, Duan H. A hybrid intelligent diagnosis approach for quick screening of Alzheimer's Disease based on multiple neuropsychological rating scales. *Computational and Mathematical Methods in Medicine*. 2015;2015:1-13. doi:10.1155/2015/258761

383. Zahlmann G, Kochner B, Ugi I, et al. Hybrid fuzzy image processing for situation assessment. A Knowledge-based system for early detection of diabetic retinopathy. *IEEE Engineering in Medicine and Biology*. 2000:76-83.

384. Zahlmann G, Scherf M, Wegner A, Obermaier M, Mertz M. Situation assessment of glaucoma using a hybrid fuzzy neural network. *IEEE Engineering in Medicine and Biology Magazine*. 2000;19(1):84-91. doi:10.1109/51.816247

385. Zamora A, Carrion C, Vazquez G, et al. A clinical decision support system can improves the quality of lipid-lowering therapy in coronary patients. In: Vol 36. European Heart Journal; 2015:285-286.

386. Zamora M, Baradad M, Amado E, et al. Characterizing chronic disease and polymedication prescription patterns from electronic health records. In: *2015 IEEE International Conference on Data Science and Advanced Analytics (DSAA)*. Campus des Cordeliers, Paris, France: IEEE; 2015:1-9. doi:10.1109/DSAA.2015.7344870

387. Zavrsnik J, Kancler K, Kokol P, Mernik M, Malcic I. The computer tool of the mitral valve prolapse determination based on automatic learning. In: *Proceedings of IEEE Symposium on Computer-Based Medical Systems (CBMS)*. Winston-Salem, NC, USA: IEEE Comput. Soc. Press; 1994:170-175. doi:10.1109/CBMS.1994.316006

388. Zavrsnik J, Kokol P, Maleiae I, Kancler K, Mernik M, Bigec M. ROSE: Decision trees, automatic learning, and their applications in cardiac medicine. *MEDINFO*. 1995;95:1688.

389. Zeuner R, Gsell U, Hubenthal M, et al. Popgen-ossa: development of an organ specific self assessment (OSSA) for interdisciplinary documentation of patient reported clinical outcomes. In: *Validation of Outcome Measures and Biomarkers*. BMJ Annals of the Rheumatic Diseases; 2017:465. doi:10.1136/annrheumdis-2017-eular.4722

390. Zhang H, Plutzky J, Shubina M, Turchin A. Continued statin prescriptions after adverse reactions and patient outcomes: a cohort study. *Annals of Internal Medicine*. 2017;167(4):221-228. doi:10.7326/M16-0838

391. Zhang J, Gajjala S, Agrawal P, et al. A computer vision pipeline for automated determination of cardiac structure and function and detection of disease by two-dimensional echocardiography. 2017. http://arxiv.org/abs/1706.07342. Accessed July 3, 2019.

392. Zhang J, Xiong H, Huang Y, Wu H, Leach K, Barnes LE. M-SEQ: Early detection of anxiety and depression via temporal orders of diagnoses in electronic health data. In: *2015 IEEE International Conference on Big Data (Big Data)*. Santa Clara, CA, USA: IEEE; 2015:2569-2577. doi:10.1109/BigData.2015.7364054

393. Zhang Q, Zhang G, Lu J, Wu D. A framework of hybrid recommender system for personalized clinical prescription. In: *2015 10th International Conference on Intelligent Systems and Knowledge Engineering (ISKE)*. Taipei, Taiwan: IEEE; 2015:189-195. doi:10.1109/ISKE.2015.98

394. Zheng K, Mei Q, Yang L, Manion FJ, Balis UJ, Hanauer DA. Voice-dictated versus typed-in clinician notes: linguistic properties and the potential implications on natural language processing. *AMIA Annual Symposium Proceedings Archive*. 2011;2011:1630-1638.

395. Zhou S-M, Fernandez-Gutierrez F, Kennedy J, et al. Defining disease phenotypes in primary care electronic health records by a machine learning approach: a case study in identifying rheumatoid arthritis. Pappalardo F, ed. *PLOS ONE*. 2016;11(5):e0154515. doi:10.1371/journal.pone.0154515

396. Zhou S-M, Muhammad RA, Sheppard S, Howe R, Lyons RA, Brophy S. Mining electronic health records to identify predictive factors associated with hospital admission for Campylobacter infections. *The Lancet*. 2017;390:S99. doi:10.1016/S0140-6736(17)33034-9

397. Zhou S-M, Rahman MA, Atkinson M, Brophy S. Mining textual data from primary healthcare records: Automatic identification of patient phenotype cohorts. In: *2014 International Joint Conference on Neural Networks (IJCNN)*. Beijing, China: IEEE; 2014:3621-3627. doi:10.1109/IJCNN.2014.6889494

398. Zhu J, Min C, Wang F. An ontology based intelligent telehealth system for long-term management of hypertension. In: Zhang Y-T, ed. *The International Conference on Health Informatics*. Vol 42. Cham: Springer International Publishing; 2014:80-83. doi:10.1007/978-3-319-03005-0_21

399. Zhuang ZY, Churilov L, Sikaris K. Uncovering the patterns in pathology ordering by Australian general practitioners: a data mining perspective. In: *Proceedings of the 39th Annual Hawaii International Conference on System Sciences (HICSS'06)*. Kauia, HI, USA: IEEE; 2006:92c-92c. doi:10.1109/HICSS.2006.513

400. Zhuang ZY, Churilov L, Burstein F, Sikaris K. Combining data mining and case-based reasoning for intelligent decision support for pathology ordering by general practitioners. *Eur J Oper Res*. 2009;195(3):662-675. doi:10.1016/j.ejor.2007.11.003

401. Zhuang ZY, Wilkin CL, Ceglowski A. A framework for an intelligent decision support system: A case in pathology test ordering. *Decis Support Sys*. 2013;55(2):476-487. doi:10.1016/j.dss.2012.10.006

402. Zhuang ZY, Amarasiri R, Churilov L, Alahakoon D, Sikaris K. Exploring the clinical notes of pathology ordering by Australian general practitioners: a text mining perspective. In: *2007 40th Annual Hawaii International Conference on System Sciences (HICSS'07)*. Waikoloa, HI: IEEE; 2007:136-136. doi:10.1109/HICSS.2007.220

403. Zortea M, Schopf TR, Thon K, et al. Performance of a dermoscopy-based computer vision system for the diagnosis of pigmented skin lesions compared with visual evaluation by experienced dermatologists. *Artificial Intelligence in Medicine*. 2014;60(1):13-26. doi:10.1016/j.artmed.2013.11.006

404. Zouridakis G, Wadhawan T, Situ N, et al. Melanoma and other skin lesion detection using smart handheld devices. In: Rasooly A, Herold KE, eds. *Mobile Health Technologies*. Vol 1256. New York, NY: Springer New York; 2015:459-496. doi:10.1007/978-1-4939-2172-0_30

405. Zvarova J, Peleska J, Hanzlicek P, Zvara K. Enhanced care of hypertensive patients using the Internet. *Medical Informatics and the Internet in Medicine*. 2002;27(3):161-168. doi:10.1080/1463923021000034651
