# Supplementary figures and images for: Health Equity in Artificial Intelligence and Primary Care Research: Protocol for a Scoping Review
Source: JMIR Res Protoc. 2021 Sep 17;10(9):e27799. doi: 10.2196/27799 (PMC8486995; doi:10.2196/27799)

## Slide 1
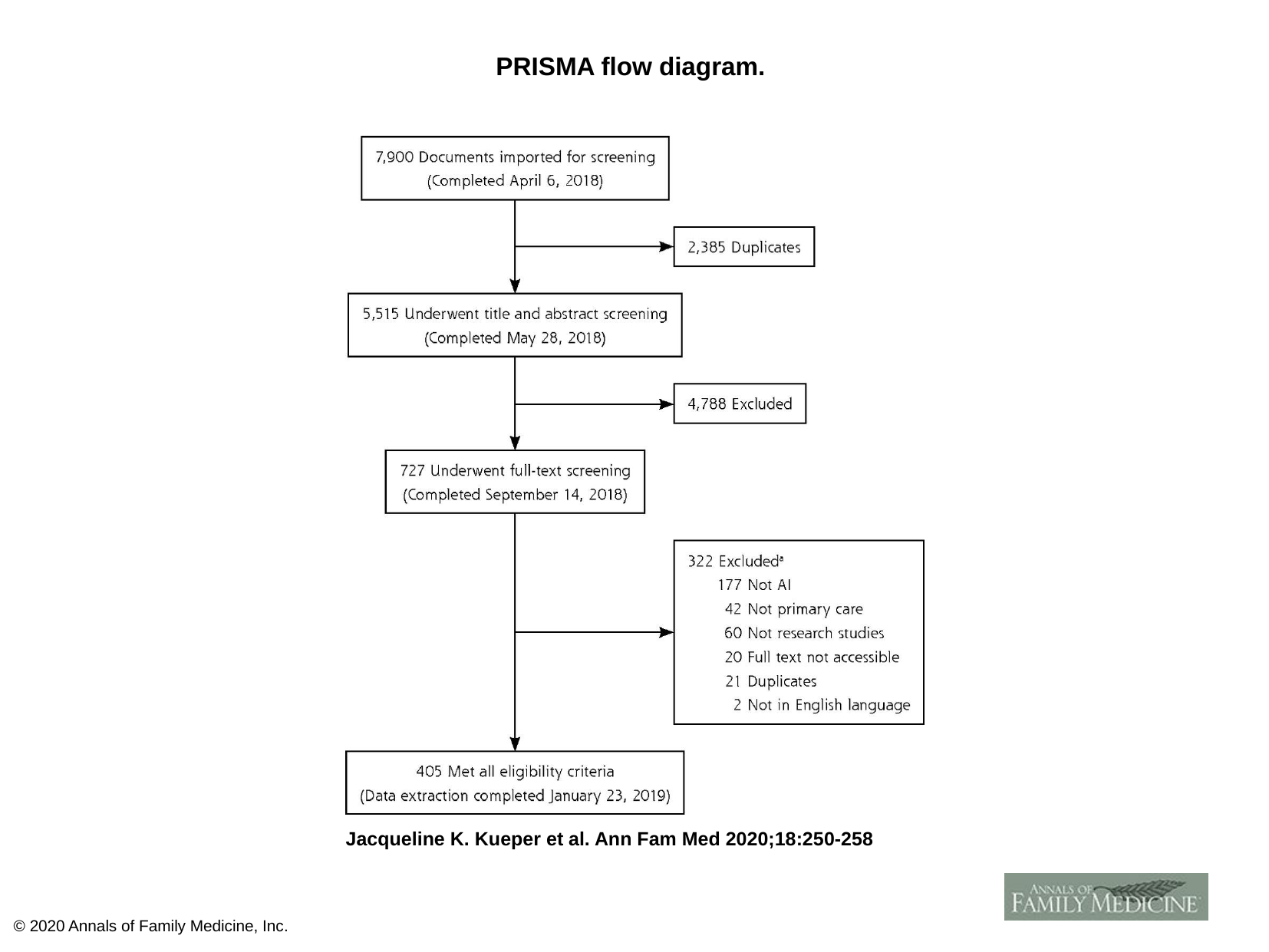

PRISMA flow diagram.
Jacqueline K. Kueper et al. Ann Fam Med 2020;18:250-258
© 2020 Annals of Family Medicine, Inc.

Supplement: Multimedia Appendix 2 [file resprot_v10i9e27799_app2.ppt]
